# Flavonoid-Rich Foods, Dementia Risk, and Interactions With Genetic Risk, Hypertension, and Depression

**DOI:** 10.1001/jamanetworkopen.2024.34136

**Published:** 2024-09-18

**Authors:** Amy Jennings, Alysha S. Thompson, Anna Tresserra-Rimbau, Joshua K. O’Neill, Claire Hill, Nicola P. Bondonno, Tilman Kühn, Aedín Cassidy

**Affiliations:** 1 The Co-Centre for Sustainable Food Systems and The Institute for Global Food Security, School of Biological Sciences, Queen’s University Belfast, Belfast, Northern Ireland, United Kingdom; 2 Department of Nutrition, Food Science and Gastronomy, XIA School of Pharmacy and Food Sciences, Nutrition and Food Safety Research Institute, University of Barcelona, Barcelona, Spain; 3 Centro de Investigación Biomédica en Red Fisiopatología de la Obesidad y la Nutrición, Instituto de Salud Carlos III, Madrid, Spain; 4 The Danish Cancer Society Research Institute, Danish Cancer Institute, Copenhagen, Denmark; 5 Nutrition & Health Innovation Research Institute, School of Medical and Health Sciences, Edith Cowan University, Perth, Western Australia, Australia; 6 Department of Nutritional Sciences, University of Vienna, Vienna, Austria; 7 Center for Public Health, Medical University of Vienna, Vienna, Austria

## Abstract

**Importance:**

A composite score of flavonoid-rich foods (flavodiet) may provide a clear public health message regarding the range of foods with the potential to lower dementia risk.

**Objective:**

To examine associations of flavodiet score and intakes of flavonoid subclasses with dementia risk according to genetic risk and presence of depression and hypertension.

**Design, Setting, and Participants:**

This prospective, population-based cohort study included dietary data from adults aged 40 to 70 years in the UK Biobank, recruited between 2006 and 2010, with a mean (SD) follow-up of 9.2 (1.5) years. The data analyses were conducted from September 1 to 30, 2023.

**Exposure:**

Flavodiet score adherence and intake of flavonoid subclasses derived from 24-hour computerized dietary assessments.

**Main Outcome and Measures:**

The main outcome was incident all-cause dementia and interactions with genetic risk, hypertension, and depressive symptoms using multivariable Cox proportional hazards regression models.

**Results:**

The sample included 121 986 participants (mean [SD] age, 56.1 [7.8] years; 55.6% female; 882 with incident dementia). Comparing the highest with lowest quintile of flavodiet score, consuming 6 additional servings per day of flavonoid-rich foods was associated with a lower risk of dementia among all participants (adjusted hazard ratio [AHR], 0.72; 95% CI, 0.57-0.89), those at high genetic risk (AHR, 0.57; 95% CI, 0.42-0.78), and those with depressive symptoms (AHR, 0.52; 95% CI, 0.33-0.81) after multivariable adjustment. The greatest risk reduction was observed in participants consuming at least 2 of the following per day: 5 servings of tea, 1 serving of red wine, and 0.5 servings of berries, compared with those who did not achieve any of these intakes (AHR, 0.62; 95% CI, 0.46-0.84). Higher intakes of flavonoid subclasses, including anthocyanins, flavan-3-ols, flavonols, and flavones, of which tea, red wine, and berries are the main contributors, supported these findings, showing inverse associations with dementia risk.

**Conclusions and Relevance:**

In this cohort study, high adherence to a flavonoid-rich diet score was associated with a lower risk of dementia, with reductions more pronounced in individuals with a high genetic risk, hypertension, and depressive symptoms. These findings suggest that simple dietary changes of increasing intakes of commonly consumed flavonoid-rich foods and drinks may lower dementia risk.

## Key Points

**Question:**

What is the association of a flavonoid-rich diet with dementia risk among UK adults?

**Findings:**

In this cohort study of 121 986 UK Biobank participants, those with the highest adherence to a flavonoid-rich diet, specifically intakes of tea, red wine, and berries, had a lower risk of dementia. Reductions were more pronounced in participants with a high genetic risk, hypertension, and depressive symptoms.

**Meaning:**

These findings suggest that increasing daily consumption of flavonoid-rich foods may lower dementia risk, especially in populations at high risk.

## Teaser

This cohort study examines the association of a flavonoid-rich diet with dementia risk based on genetic predisposition and presence of hypertension and depressive symptoms.

## Introduction

The worldwide prevalence of dementia continues to increase, with estimations of more than 50 million people living with dementia in 2020 and projections of 10 million new cases each year as population aging continues to accelerate.^1^ Currently, there is no effective treatment for dementia, so preventive interventions to improve health and quality of life and reduce social and economic costs, currently estimated at £39.4 billion in the UK alone,^2^ are a major public health priority.

While nonmodifiable risk factors, particularly age and genetics, contribute substantially to the development of dementia, evidence from cohort studies has shown that modifiable risk factors, such as diet, play an important role in prevention. Higher adherence to plant-based diets has been associated with a 21% lower risk of developing cognitive disorders and a 40% lower risk of Alzheimer disease,^3^ with diets rich in healthy plant-based foods showing the strongest associations with lower dementia risk.^4^ Flavonoids, a group of polyphenolic compounds found in plant-derived foods and beverages, such as fruits, vegetables, tea, red wine, and dark chocolate, have also been associated with a lower risk of dementia.^5,6^ There is evidence that higher intakes of berries and tea specifically are associated with a lower risk of dementia and cognitive decline.^7,8,9^ Mechanisms proposed for the neuroprotective influence of dietary flavonoids and their downstream metabolites include reducing neuroinflammation^10^; improving cerebrovascular blood flow, with many flavonoid metabolites able to permeate the blood-brain barrier and mediate the microbiome-gut-brain axis^11^; and modulating major neuronal signal transduction pathways associated with synaptic plasticity.^12^ Flavonoids have also been associated with risk factors for dementia, including hypertension and depression. Studies have reported inverse associations between flavonol, flavone, and flavanone intakes and depression risk.^13^ Furthermore, higher intakes of anthocyanins, polymers, and, specifically, the proanthocyanidin component of the flavan-3-ol polymer class have been associated with lower blood pressure.^14^

Identifying flavonoid-rich foods associated with improved health outcomes may aid in formulating dietary recommendations valuable for both intervention research and public health initiatives. As the major food sources of flavonoids are similar in different regions,^15,16^ developing a composite score of flavonoid-rich foods may provide an opportunity to deliver a clear public health message on the range of foods with the potential to lower dementia risk.^17^

The aims of this study were therefore to investigate the associations between (1) our novel flavodiet score and (2) intakes of flavonoid subclasses and dementia risk. In addition, we examined these associations according to genetic risk for dementia and presence of depression and hypertension. We hypothesized that higher intakes of berries and tea and, subsequently, intakes of anthocyanins and flavan-3-ols would be associated with lower dementia risk.

## Methods

This cohort study uses data from the UK Biobank, a large, ongoing, multicenter, population-based prospective cohort study of more than 500 000 participants recruited between 2006 and 2010.^18^ Ethical approval for the study was provided by the North West Multi-Centre Research Ethics Committee, and all participants provided written informed consent. This study follows the Strengthening the Reporting of Observational Studies in Epidemiology (STROBE) reporting guideline.

Full details of the design and methods have previously been published.^19^ Briefly, male and female participants aged 40 to 70 years were recruited from the UK using National Health Service patient registers. Participants visited 1 of 22 regional assessment centers where they completed a touchscreen questionnaire and a series of physical and biological assessments.

The current study included participants aged 40 to 70 years with appropriate dietary data defined as at least 2 dietary assessments with plausible energy intakes (800-4200 kcal/d for males or 600-3500 kcal/d for females) and complete key covariate data. Participants who withdrew consent before April 25, 2023, or had a diagnosis of dementia prior to the end of the dietary assessment period were excluded (eFigure 1 in Supplement 1).

### Dementia Outcome Ascertainment

All-cause incident dementia cases were ascertained using data linkage to hospital inpatient records and death registries. Participants with a primary or secondary diagnosis of dementia were identified from hospital records or underlying or contributory cause of death from death registries using relevant *International Classification of Diseases, Ninth Revision* and *International Statistical Classification of Diseases, Tenth Revision* codes (eTable 1 in Supplement 1). The censoring dates used for death data and hospital inpatient data were March 31, 2021, for England and Scotland and February 28, 2018, for Wales. Follow-up time was calculated from the date of last dietary assessment until either the date of first dementia diagnosis, death, loss to follow-up, or censoring date, whichever was the earliest.

### Dietary Assessment

The Oxford WebQ is a validated dietary questionnaire used to estimate the frequency of consumption of 206 foods and 32 beverages over the previous 24-hour period.^20^ Participants recruited between 2009 and 2012 completed the Oxford WebQ on up to 5 separate occasions.^21^ Data for all dietary components were averaged across all available time points after excluding time points where energy intakes were implausible. Intakes of foods estimated from 24-hour dietary recalls were previously significantly associated with urinary flavonoid biomarkers.^22^

Flavonoid values were assigned to each of the foods and beverages listed in the Oxford WebQ. For recipes, a value for each ingredient in the dishes was assigned using data from the US Department of Agriculture.^23,24,25^ Details on the flavonoid subclasses assessed are provided in eMethods 1 in Supplement 1. A flavodiet score was calculated at each time point by summing intakes (in servings per day) of the key contributors to each flavonoid subclass, including tea (black and green), red wine, apples, berries, grapes, oranges, grapefruit, sweet peppers, onions, and dark chocolate, and the cumulative average was calculated using data from time points with plausible energy intakes. The key contributors were determined as the 3 foods that contributed the highest percentage to intakes of each flavonoid subclass (excluding fruit juices), and dark chocolate was included as it is typically high in flavan-3-ols (eTable 2 in Supplement 1). The healthful plant-based diet index (hPDI) was derived from 17 food groups as previously described^26^ (eMethods 2 in Supplement 1).

### Genetic Risk and Covariates

Details on genotyping and construction of a polygenic risk score (PRS) are provided in eMethods 3 in Supplement 1. Participants were defined as being at high genetic risk for dementia if they were carriers of the apolipoprotein E (APOE) ε4 genotype or in the highest quintile of the Alzheimer disease–related PRS. Full information on the covariates used in this study are provided in eMethods 4 in Supplement 1.

### Statistical Analysis

The data analyses were conducted from September 1 to 30, 2023. Participant characteristics at baseline are presented as means with standard deviations or medians with IQRs for continuous variables and as counts with percentages for categorical variables, stratified by dementia status and quintiles of the flavodiet score. Incidence rates of dementia were calculated by dividing the number of cases by the total number of person-years at risk. Kaplan-Meier curves of time to dementia diagnosis were plotted, censoring for death, loss to follow-up, or censoring date and adjusted for sex and age at last dietary assessment. An analysis was also conducted for (1) participants at high genetic risk of dementia, defined as being a carrier of the APOE ε4 genotype or in the highest quintile of PRS; (2) participants with hypertension at baseline; and (3) participants with depressive symptoms at baseline. Multivariable Cox proportional hazards regression models were used, with age (at last dietary assessment) as the underlying timescale, to determine the associations between incident all-cause dementia and the flavodiet score and intakes of total flavonoids and subclasses in all participants and the defined subsets. The potential interaction between genetic risk (high vs low), hypertension (yes vs no), and depressive symptoms (yes vs no) and the flavodiet score and intakes of total flavonoids and subclasses on dementia risk was tested by including an interaction term in the models with all participants.

To assess whether any individual components of the flavodiet score drove the observed associations, we repeated the analyses after sequentially removing each food from the final score. After identifying the foods in the flavodiet score significantly associated with lower risk, we grouped participants according to the number of foods for which intake was defined as ideal (defined as the participants’ median intake in the top quintile of all the foods) and analyzed dementia risk.

We also examined the possibly nonlinear association between the flavodiet score and dementia by restricted cubic splines with 4 knots (fifth, 35th, 65th, and 95th percentiles), with the median value of the flavodiet score in the lowest quintile as the reference. We compared this model to the model with the linear term using the likelihood ratio test, with both models fully adjusted for all covariates.

All models were adjusted for sex (male or female), socioeconomic status (Townsend deprivation index categorized as low [quintile 1], moderate [quintiles 2-4], and high [quintile 5] deprivation), highest level of education (college, vocational, upper secondary, lower secondary, none, or unknown), self-selected race and ethnicity (Asian, Black, Chinese, White, multiracial, other, or not answered [included as disparities in dementia risk have been found^27^]), current smoking status (yes or no), typical sleep duration (≤6 hours, 7-8 hours, >8 hours, or not answered), physical activity (total excess metabolic equivalents, in quintiles), body mass index (calculated as weight in kilograms divided by height in meters squared), family history of dementia (yes or no), history of stroke (yes or no), postmenopausal status (yes, no, male, or unknown), number of medications taken (1-3, 4-6, 7-9, or ≥10), number of chronic health conditions (1-2, 3, 4, or ≥5), number of dietary assessments completed with plausible energy intakes (2, 3, 4, or 5), hPDI (score, in quintiles), alcohol intake not from red wine (grams per day, in quintiles), energy intake (kilocalories per day, in quintiles), and fat intake (grams per day, in quintiles). The adjusted models were stratified by region (London, North West England, North East England, Yorkshire, West Midlands, East Midlands, South East England, South West England, Scotland, and Wales). Models stratifying participants by genetic risk of dementia were additionally adjusted for third-degree relatedness of individuals in the sample. We also fit a Cox least absolute shrinkage and selection operator (LASSO) model for the primary analyses (flavodiet score and dementia risk in all participants) with variables automatically selected using 10-fold cross validation. Estimated hazard ratios (HRs) were calculated using the postselection coefficients of the unstandardized variables for model comparison with the fully adjusted model.

We conducted several sensitivity analyses using the same models. These analyses included (1) participants aged 60 years or older at baseline; (2) participants with more than 5 years of follow-up; (3) participants with no history of stroke; (4) participants with a high genetic risk for dementia whose reported race was White, as the PRS was defined for individuals of European ancestry; (5) adjustment for individual food components previously associated with dementia risk (ie, red and processed meats, oily fish, coffee, and foods and drinks high in sugar) and not the hPDI; (6) participants living in areas of high socioeconomic deprivation or with low levels of education; and (7) participants with low levels of physical activity.

Proportional hazards assumptions were not violated when assessed for the primary analyses using Schoenfeld residuals (*P* > .05). Multicollinearity was checked by examining the correlation matrix of the estimated coefficients from the primary model; all were less than 0.10. A 2-sided *P* < .05 was considered statistically significant by multivariable Cox proportional hazards regression. Statistical analyses were performed using Stata, version 18 software (StataCorp LLC).

## Results

The dietary intakes and demographic characteristics of 121 986 participants (mean [SD] age, 56.1 [7.8] years; 55.6% female and 44.4% male; 0.9% Asian, 0.8% Black, 0.2% Chinese, 96.6% White, 0.5% multiracial, 0.6% other, and 0.3% unknown race and ethnicity) are shown in Table 1. The characteristics of the participants included and excluded from the analysis were comparable, although those included had completed higher levels of education (eTable 3 in Supplement 1). During a median follow-up of 9.4 years (IQR, 9.3-9.8 years) accounting for 1 121 840 person-years, there were 882 cases of incident dementia. Kaplan-Meier dementia-free survival curves stratified by subsets are shown in eFigure 2 in Supplement 1.

**Table 1.** Dietary Intake and Baseline Characteristics by Quintile of Flavodiet Score Among UK Biobank Participants (N = 121 986)

Characteristic	Value, median (IQR)				
	Quintile 1 (lowest) (n = 23 264)	Quintile 2 (n = 23 944)	Quintile 3 (n = 24 004)	Quintile 4 (n = 24 605)	Quintile 5 (highest) (n = 26 169)
**Dietary**					
Flavanones, mg/d	11.2 (2.5-26.9)	15.9 (4.7-32.1)	17.9 (5.4-34.8)	18.8 (6.3-36.6)	24.9 (9.6-45)
Anthocyanins, mg/d	9.4 (3.2-22.2)	17.7 (6.2-34.3)	20.2 (7.5-38.4)	24.2 (9.8-44)	35.9 (16.4-60.8)
Flavan-3-ols, mg/d	33.8 (18.6-64.6)	116.1 (72.6-150.5)	170.8 (129.7-208.4)	222.0 (176.3-264.7)	287.5 (233.5-336.0)
Flavonols, mg/d	13.1 (8.5-18.0)	24.1 (18.8-29.1)	32.3 (26.7-37.5)	40.3 (33.9-46.0)	50.8 (43.3-58.0)
Flavones, mg/d	0.5 (0.3-0.9)	0.8 (0.4-1.2)	0.8 (0.5-1.3)	1.0 (0.6-1.6)	1.3 (0.8-2.1)
Polymers, mg/d	149.8 (89.6-235.1)	384.5 (253.7-489.8)	560.3 (418.8-669.6)	721.1 (554.9-849.2)	917.7 (718.4-1071.3)
Proanthocyanidins, mg/d^a^	150.5 (101.2-202.9)	255.6 (210.9-309.4)	329.0 (283.2-384.3)	400.0 (350.1-459.6)	508.4 (441.0-602.4)
Total flavonoids, mg/d	243.9 (156.3-356.8)	579.4 (426.1-704.0)	825.3 (669.8-950.8)	1053.2 (874.6-1196.8)	1350.3 (1135.8-1523.2)
Flavodiet score^b^	1.4 (0.8-1.9)	3.0 (2.7-3.4)	4.3 (4.0-4.5)	5.5 (5.1-5.8)	7.1 (6.5-8.0)
Flavodiet score (tea capped at 4 cups), servings/d	1.4 (0.8-1.9)	3.0 (2.7-3.4)	4.2 (4.0-4.5)	5.1 (4.8-5.5)	6.4 (5.7-7.1)
Tea	0.0 (0.0-1.0)	1.8 (1.0-2.5)	2.8 (2.0-3.5)	3.7 (3.0-4.5)	4.8 (4.0-5.6)
Red wine	0.0 (0.0-0.0)	0.0 (0.0-0.5)	0.0 (0.0-0.5)	0.0 (0.0-0.6)	0.0 (0.0-1.0)
Apples	0.0 (0.0-0.3)	0.0 (0.0-0.5)	0.3 (0.0-0.6)	0.3 (0.0-0.7)	0.5 (0.0-1.0)
Grapes	0.0 (0.0-0.0)	0.0 (0.0-0.2)	0.0 (0.0-0.3)	0.0 (0.0-0.3)	0.0 (0.0-0.5)
Dark chocolate	0.0 (0.0-0.0)	0.0 (0.0-0.0)	0.0 (0.0-0.0)	0.0 (0.0-0.0)	0.0 (0.0-0.0)
Berries	0.0 (0.0-0.0)	0.0 (0.0-0.3)	0.0 (0.0-0.3)	0.0 (0.0-0.3)	0.0 (0.0-0.5)
Onions	0.0 (0.0-0.1)	0.0 (0.0-0.2)	0.0 (0.0-0.2)	0.1 (0.0-0.3)	0.1 (0.0-0.3)
Oranges	0.0 (0.0-0.2)	0.0 (0.0-0.5)	0.0 (0.0-0.5)	0.1 (0.0-0.6)	0.5 (0.0-1.0)
Peppers	0.0 (0.0-0.0)	0.0 (0.0-0.0)	0.0 (0.0-0.1)	0.0 (0.0-0.1)	0.0 (0.0-0.2)
Grapefruit	0.0 (0.0-0.0)	0.0 (0.0-0.0)	0.0 (0.0-0.0)	0.0 (0.0-0.0)	0.0 (0.0-0.0)
Energy, kcal/d	1924.5 (1630.2-2257.4)	1957.6 (1674.1-2265.4)	2017.3 (1734.4-2338.9)	2021.4 (1746.7-2333.6)	2107.8 (1809.7-2443.7)
Fat, g/d	68.5 (54.2-84.9)	69.1 (55.1-84.9)	70.9 (56.8-86.7)	70.8 (56.6-86.5)	72.3 (57.5-89.3)
Alcohol not from red wine, g/d	6.0 (0.0-18.5)	6.0 (0.0-17.1)	5.7 (0.0-17.1)	4.5 (0.0-14.2)	3.4 (0.0-12.3)
hPDI score	51.0 (47.0-55.0)	52.0 (48.0-57.0)	53.5 (49.0-58.0)	55.0 (51.0-59.0)	57.0 (53.0-62.0)
Dietary assessment completion, d	3.0 (2.0-4.0)	3.0 (2.0-4.0)	3.0 (2.0-4.0)	3.0 (2.0-4.0)	3.0 (2.0-4.0)
**Demographic**					
Age, y	55.0 (48.0-61.0)	57.0 (49.0-62.0)	58.0 (50.0-63.0)	58.0 (51.0-63.0)	58.0 (52.0-63.0)
Duration of follow-up, y	9.4 (9.3-9.8)	9.4 (9.3-9.8)	9.4 (9.3-9.8)	9.4 (9.3-9.8)	9.4 (9.3-9.8)
Physical activity, excess METs	1023.0 (413.0-2163.0)	1130.0 (492.5-2238.0)	1182.5 (517.5-2302.0)	1204.0 (543.0-2362.5)	1334.0 (606.0-2583.0)
BMI	26.7 (24.0-30.0)	26.0 (23.5-29.0)	26.0 (23.6-28.8)	25.7 (23.4-28.6)	25.8 (23.4-28.6)
Sex, No. (%)					
Female	12 556 (54.0)	14 481 (60.5)	12 037 (50.1)	14 794 (60.1)	13 998 (53.5)
Male	10 708 (46.0)	9463 (39.5)	11 967 (49.9)	9811 (39.9)	12 171 (46.5)
Race and ethnicity, No. (%)					
Asian	263 (1.1)	303 (1.3)	265 (1.1)	168 (0.7)	116 (0.4)
Black	345 (1.5)	230 (1.0)	170 (0.7)	105 (0.4)	80 (0.3)
Chinese	71 (0.3)	74 (0.3)	56 (0.2)	55 (0.2)	54 (0.2)
White	22 186 (95.4)	22 968 (95.9)	23 193 (96.6)	23 978 (97.5)	25 593 (97.8)
Multiracial	158 (0.7)	143 (0.6)	132 (0.5)	121 (0.5)	97 (0.4)
Other^c^	173 (0.7)	167 (0.7)	125 (0.5)	108 (0.4)	125 (0.5)
Unknown	68 (0.3)	59 (0.2)	63 (0.3)	70 (0.3)	104 (0.4)
Townsend deprivation index, No. (%)					
Low	4204 (18.1)	4750 (19.8)	4914 (20.5)	5160 (21.0)	5407 (20.7)
Moderate	13 528 (58.1)	14 276 (59.6)	14 443 (60.2)	15 003 (61.0)	15 904 (60.8)
High	5532 (23.8)	4918 (20.5)	4647 (19.4)	4442 (18.1)	4858 (18.6)
Education level, No. (%)					
None	1584 (6.8)	1410 (5.9)	1502 (6.3)	1600 (6.5)	1635 (6.2)
Lower secondary	5960 (25.6)	5644 (23.6)	5350 (22.3)	5570 (22.6)	5400 (20.6)
Upper secondary	3273 (14.1)	3256 (13.6)	3141 (13.1)	3308 (13.4)	3440 (13.1)
Vocational	1240 (5.3)	1058 (4.4)	1204 (5.0)	1094 (4.4)	1349 (5.2)
College	11 141 (47.9)	12 507 (52.2)	12 744 (53.1)	12 980 (52.8)	14 281 (54.6)
Unknown	66 (0.3)	69 (0.3)	63 (0.3)	53 (0.2)	64 (0.2)
Current smoker, No. (%)					
No	20 964 (90.1)	22 346 (93.3)	22 546 (93.9)	23 196 (94.3)	24 552 (93.8)
Yes	2300 (9.9)	1598 (6.7)	1458 (6.1)	1409 (5.7)	1617 (6.2)
Sleep duration, , No. (%), h					
7-8	16 228 (69.8)	17 310 (72.3)	17 530 (73.0)	18 004 (73.2)	18 919 (72.3)
≤6	5451 (23.4)	5087 (21.2)	5003 (20.8)	5069 (20.6)	5608 (21.4)
>8	1533 (6.6)	1513 (6.3)	1436 (6)	1499 (6.1)	1594 (6.1)
Unknown	52 (0.2)	34 (0.1)	35 (0.1)	33 (0.1)	48 (0.2)
Family history of dementia, No. (%)					
No	20 454 (87.9)	20 736 (86.6)	20 819 (86.7)	21 131 (85.9)	22 541 (86.1)
Yes	2810 (12.1)	3208 (13.4)	3185 (13.3)	3474 (14.1)	3628 (13.9)
Stroke history, No. (%)					
No	22 629 (97.3)	23 411 (97.8)	23 409 (97.5)	24 079 (97.9)	25 525 (97.5)
Yes	635 (2.7)	533 (2.2)	595 (2.5)	526 (2.1)	644 (2.5)
Postmenopausal status, No. (%)					
No	3897 (16.8)	3906 (16.3)	3088 (12.9)	3522 (14.3)	3199 (12.2)
Yes	6673 (28.7)	8420 (35.2)	7201 (30.0)	9051 (36.8)	8687 (33.2)
Male	10 708 (46)	9463 (39.5)	11 967 (49.9)	9811 (39.9)	12 171 (46.5)
Unknown	1986 (8.5)	2155 (9.0)	1748 (7.3)	2221 (9.0)	2112 (8.1)
No. of medications taken, No. (%)					
0	7269 (31.2)	7430 (31.0)	7665 (31.9)	7545 (30.7)	8068 (30.8)
1-3	10 807 (46.5)	11 350 (47.4)	11 128 (46.4)	11 563 (47.0)	12 075 (46.1)
4-6	3732 (16.0)	3817 (15.9)	3837 (16.0)	4143 (16.8)	4468 (17.1)
7-9	1040 (4.5)	1003 (4.2)	1017 (4.2)	1009 (4.1)	1163 (4.4)
>10	416 (1.8)	344 (1.4)	357 (1.5)	345 (1.4)	395 (1.5)
No. of chronic conditions, No. (%)					
0	8347 (35.9)	8790 (36.7)	8557 (35.6)	8586 (34.9)	8898 (34.0)
1	7597 (32.7)	7826 (32.7)	7797 (32.5)	8068 (32.8)	8553 (32.7)
2	4111 (17.7)	4307 (18.0)	4521 (18.8)	4582 (18.6)	5047 (19.3)
>3	3209 (13.8)	3021 (12.6)	3129 (13.0)	3369 (13.7)	3671 (14.0)
APOE ε4 carrier, No. (%)					
No	16 430 (72.2)	17 025 (72.7)	16 999 (72.3)	17 341 (71.9)	18 291 (71.4)
Yes	6313 (27.8)	6397 (27.3)	6501 (27.7)	6764 (28.1)	7342 (28.6)
Genetic risk category, No. (%)					
Low	4562 (20.1)	4784 (20.4)	4605 (19.6)	4848 (20.1)	5073 (19.8)
Medium	13 699 (60.3)	14 011 (59.8)	14 185 (60.4)	14 422 (59.9)	15 295 (59.7)
High	4470 (19.7)	4620 (19.7)	4701 (20)	4825 (20)	5254 (20.5)
Genetic kinship, No. (%)					
At least 1 relative identified	6308 (27.1)	6340 (26.5)	6452 (26.9)	6938 (28.2)	7327 (28.0)
No kinship found	16 477 (70.8)	17 116 (71.5)	17 073 (71.1)	17 191 (69.9)	18 334 (70.1)
Unknown	479 (2.1)	488 (2.0)	479 (2.0)	476 (1.9)	508 (1.9)
Depressive symptoms, No. (%)					
No	11 (<1.0)	16 (0.1)	10 (<1.0)	11 (<1.0)	17 (0.1)
Yes	23 253 (>99.9)	23 928 (99.9)	23 994 (>99.9)	24 594 (>99.9)	26 152 (99.9)
Hypertension, No. (%)					
No	13 220 (56.8)	13 558 (56.6)	13 500 (56.2)	13 769 (56)	14 240 (54.4)
Yes	10 043 (43.2)	10 385 (43.4)	10 503 (43.8)	10 834 (44.0)	11 929 (45.6)

Abbreviations: APOE, apolipoprotein E; BMI, body mass index (calculated as weight in kilograms divided by height in meters squared); hPDI, healthful plant-based diet index.

^a^ Proanthocyanidins are also included in the polymer subclass.

^b^ Flavodiet score was calculated by summing intakes (in servings per day) of tea (black and green), red wine, apples, berries, grapes, oranges, grapefruit, sweet peppers, onions, and dark chocolate.

^c^ Other race and ethnicity was self-selected and includes no additional information.

Participants reported a median 4.3 (IQR, 2.8-5.9) servings per day of flavonoid-rich foods, of which a median 2.7 (IQR, 1.0-4.0) servings per day were from tea (eTable 4 in Supplement 1). Comparing participants in the highest vs lowest quintiles of the flavodiet score, there was a mean (SE) difference of 6.2 (0.01) servings per day of flavonoid-rich foods. Foods in the flavodiet score contributed to 85.8% of total flavonoid intake and ranged from 47.4% (flavanone) to 91.1% (flavan-3-ol) of intake of the flavonoid subclasses (eTable 2 in Supplement 1). Participants in the highest vs lowest quintiles of the flavodiet score were more physically active (median, 1334 [IQR, 606-2583] vs 1023 [IQR, 413-2163] excess METs), had a lower BMI (median, 25.8 [IQR, 23.4-28.6] vs 26.7 [IQR, 24.0-30.0]), and experienced less socioeconomic deprivation (18.6% vs 23.8%) (Table 1).

**Figure 1.**
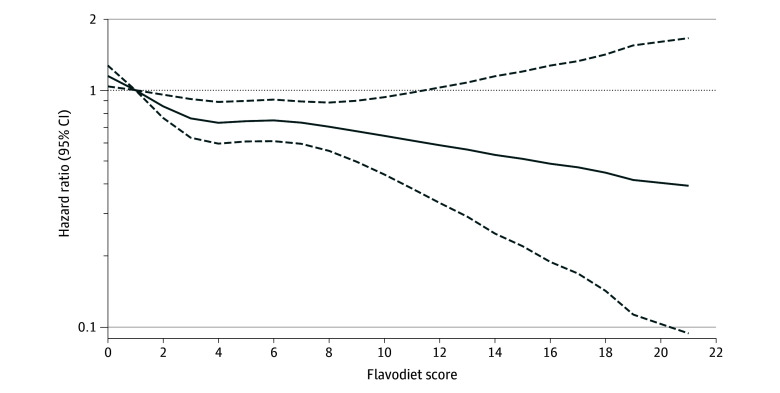
Risk of Dementia by Flavodiet Score in UK Biobank Participants Values are adjusted based on cubic splines (N = 121 986 [882 with incident dementia]). Flavodiet score calculation and model adjustment are described in the Methods.

Among all participants, those with the highest flavodiet scores (quintile 5) had a lower risk of dementia compared with those with the lowest flavodiet scores (quintile 1), after multivariable adjustments (adjusted HR [AHR], 0.72; 95% CI, 0.57-0.89; *P* for trend = .03) (Table 2). The LASSO model found that 11 of the 17 variables with nonzero coefficients for inclusion and the coefficients for the association between the flavodiet score and dementia risk were not markedly changed compared with the fully adjusted model (estimated HR per quintile of flavodiet score in LASSO model, 0.95) (eTable 5 in Supplement 1) vs 0.94 in Cox proportional hazards model. The cubic spline model showed associations with a lower risk for dementia at intakes up to 11 portions of flavonoid-rich foods per day (Figure 1), although there was no evidence that a restricted cubic spline model fitted the data better (likelihood ratio test *P* = .99).

**Table 2.** Risk of Dementia by Quintiles of Flavodiet Score and Flavonoid Subclass Intake Among UK Biobank Participants^a^

Intake component	Quintile 1 (lowest)	Quintile 2	Quintile 3	Quintile 4	Quintile 5 (highest)	*P* value for trend
**Flavodiet score, points** ^b^						
Intake, median (IQR)	1.4 (0.8-1.9)	3 (2.7-3.4)	4.3 (4.0-4.5)	5.5 (5.1-5.8)	7.3 (0.0-8.1)	.03
No. of participants (incident cases of dementia)	23 264 (173)	23 944 (161)	24 004 (173)	24 605 (194)	26 169 (181)	
AHR (95% CI)	1 [Reference]	0.79 (0.64-0.98)	0.79 (0.64-0.98)	0.86 (0.69-1.06)	0.72 (0.57-0.89)	
**Flavanones, mg/d**						
Intake, median (IQR)	1.1 (0.4-2.2)	7.2 (5.2-9.5)	17.9 (14.7-20.4)	31 (27.0-35.7)	53.9 (0.0-67.4)	.62
No. of participants (incident cases of dementia)	24 399 (177)	24 396 (171)	24 397 (165)	24 397 (171)	24 397 (198)	
AHR (95% CI)	1 [Reference]	0.94 (0.76-1.16)	0.87 (0.70-1.08)	0.87 (0.71-1.08)	0.97 (0.78-1.19)	
**Anthocyanins, mg/d**						
Intake, median (IQR)	2.6 (1.4-3.8)	9.3 (7.1-12)	20.5 (17.6-23.8)	35.7 (31.0-40.3)	64.1 (0.0-81.2)	.03
No. of participants (incident cases of dementia)	24 398 (160)	24 397 (173)	24 397 (179)	24 397 (186)	24 397 (184)	
AHR (95% CI)	1 [Reference]	0.9 (0.72-1.11)	0.86 (0.69-1.07)	0.83 (0.67-1.04)	0.78 (0.62-0.97)	
**Flavan-3-ols, mg/d**						
Intake, median (IQR)	31.5 (18.7-47.1)	103.1 (85.1-120.4)	166.1 (150.7-180.6)	228.5 (211.4-247.1)	324.3 (0.0-378.1)	.002
No. of participants (incident cases of dementia)	24 398 (193)	24 397 (171)	24 397 (188)	24 397 (163)	24 397 (167)	
AHR (95% CI)	1 [Reference]	0.80 (0.65-0.98)	0.83 (0.67-1.01)	0.71 (0.57-0.88)	0.72 (0.58-0.89)	
**Flavonols, mg/d**						
Intake, median (IQR)	11.8 (8.2-14.8)	22.7 (20.3-25.1)	31.8 (29.6-33.9)	40.9 (38.4-43.5)	53.6 (0.0-59.5)	.01
No. of participants (incident cases of dementia)	24 398 (193)	24 397 (175)	24 397 (149)	24 397 (186)	24 397 (179)	
AHR (95% CI)	1 [Reference]	0.81 (0.66-1.00)	0.65 (0.52-0.80)	0.78 (0.64-0.96)	0.73 (0.59-0.91)	
**Flavones, mg/d**						
Intake, median (IQR)	0.2 (0.1-0.3)	0.5 (0.5-0.6)	0.9 (0.8-1.0)	1.3 (1.2-1.4)	2.2 (0.0-2.8)	.03
No. of participants (incident cases of dementia)	24 398 (181)	24 397 (164)	24 397 (189)	24 397 (187)	24 397 (161)	
AHR (95% CI)	1 [Reference]	0.79 (0.63-0.97)	0.87 (0.71-1.08)	0.84 (0.68-1.05)	0.71 (0.56-0.90)	
**Polymers, mg/d**						
Intake, median (IQR)	131.7 (85.0-175.0)	319.2 (268.5-372.1)	520.0 (472.1-566.5)	713.3 (661.4-766.9)	978.3 (0.0-1086.4)	.049
No. of participants (incident cases of dementia)	24 398 (169)	24 397 (168)	24 397 (195)	24 397 (185)	24 397 (165)	
AHR (95% CI)	1 [Reference]	0.87 (0.70-1.08)	0.97 (0.79-1.20)	0.88 (0.71-1.08)	0.77 (0.62-0.97)	
**Proanthocyanidins, mg/d** ^c^						
Intake, median (IQR)	144.7 (101.2-178.1)	253.5 (230.2-275.0)	335.8 (316.1-355.9)	420.7 (397.8-446.3)	564.2 (0.0-665.8)	.049
No. of participants (incident cases of dementia)	24 398 (176)	24 397 (167)	24 397 (171)	24 397 (181)	24 397 (187)	
AHR (95% CI)	1 [Reference]	0.83 (0.67-1.03)	0.79 (0.64-0.99)	0.79 (0.64-0.98)	0.78 (0.63-0.98)	
**Total flavonoids, mg/d**						
Intake, median (IQR)	234.7 (157.6-305.9)	526.6 (452.4-597.8)	792.8 (729.6-853.4)	1048.0 (980.3-1118.9)	1399.7 (0.0-1538.8)	.008
No. of participants (incident cases of dementia)	24 398 (188)	24 397 (156)	24 397 (189)	24 397 (179)	24 397 (170)	
AHR (95% CI)	1 [Reference]	0.73 (0.59-0.91)	0.83 (0.67-1.02)	0.75 (0.61-0.93)	0.71 (0.57-0.88)	

Abbreviation: AHR, adjusted hazard ratio.

^a^ Total number of participants, 121 986; number of participants with incident dementia, 882.

^b^ Flavodiet score was calculated by summing intakes (in servings per day) of tea (black and green), red wine, apples, berries, grapes, oranges, grapefruit, sweet peppers, onions, and dark chocolate.

^c^ Proanthocyanidins are also included in the polymer subclass. Model adjustment details are provided in the Methods.

A higher flavodiet score in which intake of tea was capped at 4 servings per day was also associated with a lower risk for dementia (quintile 5 vs 1: AHR, 0.74; 95% CI, 0.60-0.93; *P* for trend = .03) (eTable 6 in Supplement 1). In a repeat analysis of the flavodiet score, where each component was sequentially removed, the flavodiet score was not associated with dementia risk after tea, red wine, and berries were removed (eTable 6 in Supplement 1). Median intakes of tea, red wine, and berries among participants in the highest quintile of all 3 foods (n = 1297) were 5 (IQR, 4.0-5.6), 0.0 (IQR, 0.0-1.0), and 0.5 (IQR, 0.0-1.0) servings per day, respectively. Participants who met at least 2 of these ideal intakes (n = 8270) had a lower dementia risk (AHR, 0.62; 95% CI, 0.46-0.84) compared with participants who did not achieve any of the intakes (n = 71 129) (eFigure 3 in Supplement 1).

**Figure 2.**
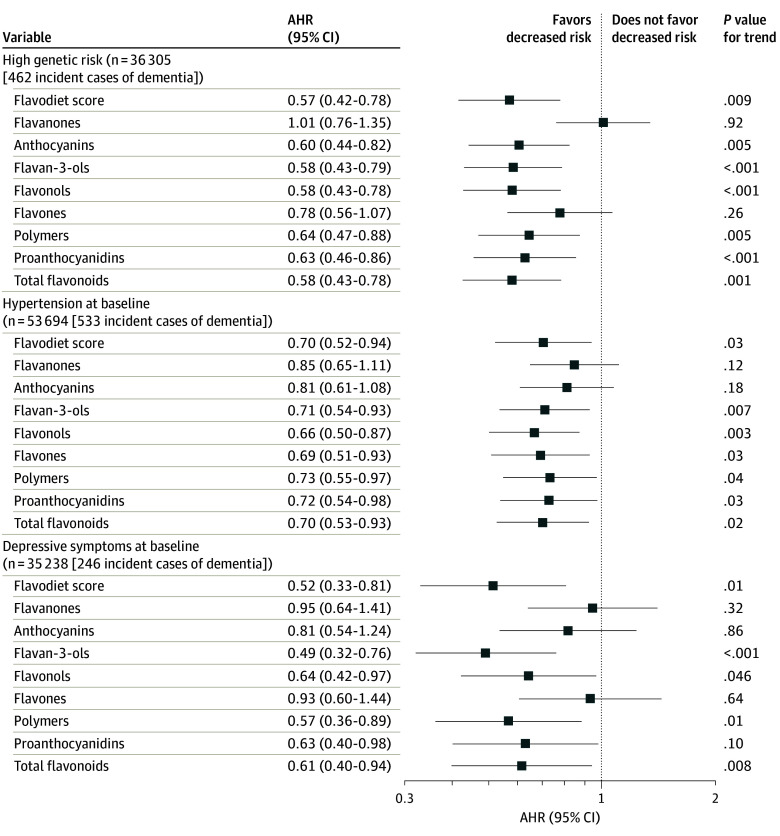
Risk of Dementia per Quintile of Flavodiet Score and Flavonoid Subclass Intake in Participants at High Genetic Risk of Dementia and With Hypertension and Depressive Symptoms at Baseline Participants at high genetic risk of dementia numbered 36 305; with hypertension, 53 694; and with depressive symptoms, 35 238. Values compare highest with lowest quintiles of intake. Flavodiet score calculation and model adjustment are described in the Methods. AHR indicates adjusted hazard ratio.

In stratified analysis, a higher flavodiet score (quintile 5 vs 1) was associated with a lower risk of dementia in participants at high genetic risk (AHR, 0.57; 95% CI, 0.42-0.78; *P* for trend = .01; *P* for interaction = .02) (Figure 2; eTable 7 in Supplement 1), with depressive symptoms (AHR, 0.52; 95% CI, 0.31-0.81; *P* for trend = .01; *P* for interaction = .01) (Figure 2; eTable 8 in Supplement 1), and with hypertension (AHR, 0.70; 95% CI, 0.52-0.94; *P* for trend = .03; *P* for interaction = .51) (Figure 2; eTable 9 in Supplement 1). No associations between the flavodiet score and dementia risk were observed in participants at low genetic risk without depressive symptoms or hypertension (eTables 7-9 in Supplement 1). Of the flavonoid subclasses, higher intakes (quintile 5 vs 1) of anthocyanins (AHR, 0.78; 95% CI, 0.62-0.97; *P* for trend = .03), flavan-3-ols (AHR, 0.72; 95% CI, 0.58-0.89; *P* for trend = .002), flavonols (AHR, 0.73; 95% CI, 0.59-0.91; *P* for trend = .01), and flavones (AHR, 0.71; 95% CI, 0.56-0.90); *P* for trend = .03) were associated with a lower risk of dementia (Table 2).

In stratified analysis, higher intakes of all the flavonoid subclasses, except for the flavanone and flavone subclasses, were associated with a lower risk of dementia in participants at high genetic risk, with no associations observed in those at low genetic risk (eTable 7 in Supplement 1). A significant interaction with genetic risk was observed for the anthocyanin (quintile 5 vs 1: AHR, 0.60; 95% CI, 0.44-0.82; *P* for trend < .001; *P* for interaction = .01) and flavonol (quintile 5 vs 1: AHR, 0.58; 95% CI, 0.43-0.78; *P* for trend < .001; *P* for interaction = .03) subclasses.

Higher intakes of flavan-3-ols (quintile 5 vs 1: AHR, 0.49; 95% CI, 0.32-0.76; *P* for trend = .001; *P* for interaction < .01) and polymers (quintile 5 vs 1: AHR, 0.57; 95% CI, 0.36-0.89; *P* for trend = .01; *P* for interaction = .04) were associated with a lower risk of dementia in participants with depressive symptoms (eTable 8 in Supplement 1). For the anthocyanin subclass, associations between higher intakes and lower dementia risk were observed in participants without depressive symptoms (quintile 5 vs 1: AHR, 0.75; 95% CI, 0.57-0.97; *P* for trend = .01) (eTable 8 in Supplement 1) or hypertension (quintile 5 vs 1: AHR, 0.69; 95% CI, 0.48-0.99; *P* for trend = .03) (eTable 9 in Supplement 1) at baseline, although no statistical interactions were observed.

Associations between higher adherence to the flavodiet score and higher intake of anthocyanins, flavan-3-ol, flavonols, and flavones with lower dementia risk were not markedly changed in sensitivity analyses of participants aged 60 years or older at baseline (eTable 10 in Supplement 1) and participants with more than 5 years of follow-up (eTable 11 in Supplement 1), no history of stroke (eTable 12 in Supplement 1), a high genetic risk whose reported race was White (eTable 13 in Supplement 1), residency in areas of high socioeconomic deprivation or low levels of education (eTable 15 in Supplement 1), and low or moderate levels of physical activity (eTable 16 in Supplement 1). Furthermore, no association was found after adjusting for individual food components and removing hPDI from the model (eTable 14 in Supplement 1).

## Discussion

The findings from this large prospective cohort study of more than 120 000 UK Biobank participants suggest that consuming 6 additional servings per day of flavonoid-rich foods, specifically tea, red wine, and berries, was associated with a lower risk of dementia, with the strongest associations observed for tea. We were also able to show, for the first time in our knowledge, that these associations were evident in participants at high genetic risk of dementia and those with modifiable risk factors, including depression and hypertension, but not in those without.

Intakes of tea have previously been associated with lower dementia risk in this cohort, with a 14% lower dementia risk in participants consuming more than 4 servings per day of tea compared with those who did not consume tea.^9^ This risk is one-half of the risk we observed for the combination of flavonoid-rich foods in the current analyses, highlighting the benefits of consuming a range of flavonoid-rich foods for lowering dementia risk. Our findings appeared to be driven by intakes of tea, berries, and red wine, with the greatest risk reduction observed in participants consuming at least 2 of the following: 5 servings per day of tea, 1 serving per day of red wine, and 0.5 servings per day of berries. These findings are supported by our analyses of flavonoid subclasses showing that the anthocyanin, flavan-3-ol, and flavone subclasses, of which tea, red wine, and berries are among the top contributors, had the strongest associations with dementia risk. Thus, modest changes to the diet could increase flavonoid levels to that required to lower dementia risk.

We found a more pronounced protective association of flavonoid-rich food with dementia risk in participants at high genetic risk of dementia. In silico studies have shown that epicatechin, a flavonoid found in tea, inhibits and disrupts the pathologic functions of APOE, including modulating the interaction with amyloid-β, the main component of amyloid plaques in the brain that are markers of Alzheimer disease.^28^ Previous human studies on the interaction between APOE genotype and flavonoid-rich foods have shown that intake of total fruit and vegetables was associated with a decreased risk of dementia with no interaction by APOE status.^29^ Likewise, more frequent consumption of fruit and vegetable juices delayed the onset of Alzheimer disease, particularly in APOE ε4 carriers, with no associations observed for tea or red wine.^30^ While APOE carrier status is one of the strongest genetic risk factors for dementia, its contribution to incident dementia is greater in younger people. In older individuals, the PRS is important due to the cumulative contribution of genes outside of the APOE region.^31^ By combining information on APOE genotype and PRSs, we offer a comprehensive measure of genetic risk that considers the biological mechanisms of dementia development identified by the polygenic component over and above APOE alone.

Another key finding of this study was that the association between flavonoid-rich food consumption and dementia risk was observed in participants with depressive symptoms at baseline. Depression activates many of the mechanistic pathways known to be targeted by flavonoids, including increased proinflammatory cytokines,^32^ reduced cerebral blood flow,^33^ and modified vascular risk factors.^34^ Several preclinical studies have shown that flavonoids, including anthocyanins and catechin, reverse depressive behavior in animal models through increased expression levels of various neurotransmitters and brain-derived neurotrophic factor.^35,36^ Conversely, we found that the associations between anthocyanin intake and dementia risk were only observed in participants without depressive symptoms at baseline. While red wine, a key contributor of anthocyanin intake, has been shown to be beneficial for both dementia and depression at moderate intakes,^37,38^ the associations among alcohol intake, depression, and dementia are complex, and future studies are needed to further understand these findings. Moreover, our population-based study could not identify whether moderate alcohol or anthocyanins in red wine underlie the observed associations. The current data on alcohol intake and dementia risk are equivocal. Several studies have suggested that moderate alcohol consumption may protect against dementia^39^; however, in a recent Mendelian randomization study, low to moderate alcohol consumption was not associated with the risk for Alzheimer disease but was associated with an earlier age at the onset of Alzheimer disease,^40^ which does not support a beneficial role of red wine or other alcoholic beverages in dementia prevention. Current UK health guidance recommends that alcohol consumption be reduced as much as possible, particularly during midlife, to minimize the risk of developing dementia.^41^

### Strengths and Limitations

This study has several strengths. It is the largest study to date to examine flavonoids and dementia risk in a well-characterized, prospective cohort with a long follow-up period. In addition, we defined genetic risk using a comprehensive measure incorporating both APOE genotype and PRSs.

The study also has several limitations. First, although we collected dietary data using a validated, repeated, 24-hour dietary assessment, which included intakes of all the key contributors to flavonoid intake and relied on ranked exposure rather than estimates of absolute intake,^20^ as with all self-reported dietary assessments, there are known measurement errors and reporting biases.^42^ Second, the number of participants not consuming flavonoid-rich foods was too small to define; thus, we were not able to examine associations between consumers and nonconsumers. Third, the findings may not be generalizable to other populations, as UK Biobank participants are generally healthier, with lower rates of obesity and smoking; experience less socioeconomic deprivation than the general population; and consume high amounts of tea. However, valid assessment of exposure-disease associations does not require participants to be representative of the general population.^43^ Fourth, use of health records to identify dementia cases may be biased, as poor cognitive ability is a factor that has been associated with loss to follow-up.^44^ Of note, a validation study investigating the accuracy of the UK Biobank dataset for dementia case ascertainment showed a high predictive value for all-cause dementia with lower accuracy for the dementia subtypes compared with clinical adjudication.^45^ Despite the large dataset, this study may have been underpowered to detect associations in certain subgroups. Finally, as with all observational studies, despite our detailed adjustment for a range of dietary and lifestyle variables, there is still the possibility of residual or unmeasured confounding from additional variables. Despite the prospective nature of the study, we cannot rule out reverse causation, as participants who developed dementia may have altered dietary preferences before the onset of symptoms.

## Conclusions

In this cohort study, higher flavonoid-rich diet scores were associated with a lower risk of dementia, with reductions most pronounced in individuals with a high genetic risk, hypertension, and depressive symptoms. Our results suggest that inclusion of flavonoid-rich foods into the daily diet may lower dementia risk, especially in populations at high risk.

## Notes

Supplement 1. **eMethods 1.** Flavonoid Subclasses **eMethods 2.** Healthy Plant-Based Diet Index **eMethods 3.** Genetic Risk **eMethods 4.** Covariate Assessment **eFigure 1.** Flowchart of Participants in the UK Biobank Study **eFigure 2.** Age- and Sex-Adjusted Kaplan-Meier Survival Curves in All Participants and Stratified by Subgroup in 121 986 Males and Females From the UK Biobank **eFigure 3.** Risk of Dementia by the Number of Components Met of Tea Intake of ≥5 Servings/d, Red Wine Intake ≥1 Serving/d, and Berry Intake ≥0.5 Servings/d in 121 986 Males and Females From the UK Biobank **eTable 1.** *International Classification of Diseases* Codes Used for Ascertainment of Dementia **eTable 2.** Percent Contribution of Flavonoid-Rich Foods and the Flavodiet Score to Intake of Flavonoid Subclasses in 121 986 Males and Females From the UK Biobank **eTable 3.** Baseline Characteristics by Inclusion Status in 502 411 Males and Females From the UK Biobank **eTable 4.** Baseline Characteristics and Dietary Intakes by Dementia Diagnosis in 121 986 Males and Females From the UK Biobank **eTable 5.** Estimated Hazard Ratios for LASSO Cox Regression Between Risk of Dementia and Flavodiet Score in 121 986 Males and Females From the UK Biobank **eTable 6.** Risk of Dementia by Quintiles of Flavodiet Score After Removing Each Food From the Final Score in 121 986 Males and Females From the UK Biobank **eTable 7.** Risk of Dementia by Quintiles of Flavodiet Score and Flavonoid Subclass Intake in 121 986 Males and Females From the UK Biobank, Stratified by Genetic Risk **eTable 8.** Risk of Dementia by Quintiles of Flavodiet Score and Flavonoid Subclass Intake in 121 105 Males and Females From the UK Biobank, Stratified by Depression Status **eTable 9.** Risk of Dementia by Quintiles of Flavodiet Score and Flavonoid Subclass Intake in 121 981 Males and Females From the UK Biobank, Stratified by Hypertension Status **eTable 10.** Risk of Dementia by Quintiles of Flavodiet Score and Flavonoid Subclass Intake in 61 719 Males and Females Aged More Than 60 Years From the UK Biobank **eTable 11.** Risk of Dementia by Quintiles of Flavodiet Score and Flavonoid Subclass Intake in 120 959 Males and Females With at Least 5 Years Follow-Up From the UK Biobank **eTable 12.** Risk of Dementia by Quintiles of Flavodiet Score and Flavonoid Subclass Intake in 119 053 Males and Females With No Reported Stroke History From the UK Biobank **eTable 13.** Risk of Dementia by Quintiles of Flavodiet Score and Flavonoid Subclass Intake in 35 110 Males and Females At High Genetic Risk of Dementia Whose Reported Ethnicity Was White From the UK Biobank **eTable 14.** Risk of Dementia by Quintiles of Flavodiet Score and Flavonoid Subclass Intake in 121 986 Males and Females From the UK Biobank Adjusted for Individual Foods Associated With Dementia Risk) **eTable 15.** Risk of Dementia by Quintiles of Flavodiet Score and Flavonoid Subclass Intake in 53 101 Males and Females From the UK Biobank Living in High Areas of Deprivation or With Low Levels of Education **eTable 16.** Risk of Dementia by Quintiles of Flavodiet Score and Flavonoid Subclass Intake in 73 200 Males and Females From the UK Biobank With Low or Moderate Levels of Physical Activity
eReferences

Supplement 2.
Data Sharing Statement

## References

[1] Prince M, Wimo A, Guerchet M, Ali GC, Wu YT, Prina M. World Alzheimer Report 2015. The Global Impact of Dementia: An Analysis of Prevalence, Incidence, Cost and Trends. Alzheimer’s Disease International; 2015.

[2] Wittenberg R, Hu B, Jagger C, . Projections of care for older people with dementia in England: 2015 to 2040. Age Ageing. 2020;49(2):264-269. doi:10.1093/ageing/afz154 31808792 PMC7047814

[3] Wu L, Sun D. Adherence to Mediterranean diet and risk of developing cognitive disorders: an updated systematic review and meta-analysis of prospective cohort studies. Sci Rep. 2017;7:41317. doi:10.1038/srep41317 28112268 PMC5256032

[4] Wu H, Gu Y, Meng G, . Quality of plant-based diet and the risk of dementia and depression among middle-aged and older population. Age Ageing. 2023;52(5):afad070. doi:10.1093/ageing/afad070 37247402

[5] Shishtar E, Rogers GT, Blumberg JB, Au R, Jacques PF. Long-term dietary flavonoid intake and risk of Alzheimer disease and related dementias in the Framingham Offspring Cohort. Am J Clin Nutr. 2020;112(2):343-353. doi:10.1093/ajcn/nqaa079 32320019 PMC7398772

[6] Bondonno CP, Bondonno NP, Dalgaard F, . Flavonoid intake and incident dementia in the Danish Diet, Cancer, and Health Cohort. Alzheimers Dement (N Y). 2021;7(1):e12175. doi:10.1002/trc2.12175 34027025 PMC8118115

[7] Agarwal P, Holland TM, Wang Y, Bennett DA, Morris MC. Association of strawberries and anthocyanidin intake with Alzheimer’s dementia risk. Nutrients. 2019;11(12):3060. doi:10.3390/nu11123060 31847371 PMC6950087

[8] Devore EE, Kang JH, Breteler MM, Grodstein F. Dietary intakes of berries and flavonoids in relation to cognitive decline. Ann Neurol. 2012;72(1):135-143. doi:10.1002/ana.23594 22535616 PMC3582325

[9] Zhang Y, Yang H, Li S, Li WD, Wang Y. Consumption of coffee and tea and risk of developing stroke, dementia, and poststroke dementia: a cohort study in the UK Biobank. PLoS Med. 2021;18(11):e1003830. doi:10.1371/journal.pmed.1003830 34784347 PMC8594796

[10] Hamsalakshmi, Alex AM, Arehally Marappa M, Joghee S, Chidambaram SB. Therapeutic benefits of flavonoids against neuroinflammation: a systematic review. Inflammopharmacology. 2022;30(1):111-136. doi:10.1007/s10787-021-00895-8 35031904

[11] Youdim KA, Qaiser MZ, Begley DJ, Rice-Evans CA, Abbott NJ. Flavonoid permeability across an in situ model of the blood-brain barrier. Free Radic Biol Med. 2004;36(5):592-604. doi:10.1016/j.freeradbiomed.2003.11.023 14980703

[12] Vauzour D, Vafeiadou K, Rice-Evans C, Williams RJ, Spencer JP. Activation of pro-survival Akt and ERK1/2 signalling pathways underlie the anti-apoptotic effects of flavanones in cortical neurons. J Neurochem. 2007;103(4):1355-1367. doi:10.1111/j.1471-4159.2007.04841.x 17961201

[13] Chang SC, Cassidy A, Willett WC, Rimm EB, O’Reilly EJ, Okereke OI. Dietary flavonoid intake and risk of incident depression in midlife and older women. Am J Clin Nutr. 2016;104(3):704-714. doi:10.3945/ajcn.115.124545 27413131 PMC4997290

[14] Jennings A, Koch M, Bang C, Franke A, Lieb W, Cassidy A. Microbial diversity and abundance of *Parabacteroides* mediate the associations between higher intake of flavonoid-rich foods and lower blood pressure. Hypertension. 2021;78(4):1016-1026. doi:10.1161/HYPERTENSIONAHA.121.17441 34420369

[15] Sebastian R, Goldman J, Moshfegh A. Dietary Intake and Sources of Flavonoids by Adults in the U.S. What We Eat in America, NHANES 2017-2018. US Department of Agriculture; 2023. 38768289

[16] Zamora-Ros R, Knaze V, Luján-Barroso L, . Differences in dietary intakes, food sources and determinants of total flavonoids between Mediterranean and non-Mediterranean countries participating in the European Prospective Investigation into Cancer and Nutrition (EPIC) study. Br J Nutr. 2013;109(8):1498-1507. doi:10.1017/S0007114512003273 22980437

[17] Bondonno NP, Liu YL, Zheng Y, . Change in habitual intakes of flavonoid-rich foods and mortality in US males and females. BMC Med. 2023;21(1):181. doi:10.1186/s12916-023-02873-z 37173745 PMC10182674

[18] Ollier W, Sprosen T, Peakman T. UK Biobank: from concept to reality. Pharmacogenomics. 2005;6(6):639-646. doi:10.2217/14622416.6.6.639 16143003

[19] Sudlow C, Gallacher J, Allen N, . UK Biobank: an open access resource for identifying the causes of a wide range of complex diseases of middle and old age. PLoS Med. 2015;12(3):e1001779. doi:10.1371/journal.pmed.1001779 25826379 PMC4380465

[20] Greenwood DC, Hardie LJ, Frost GS, . Validation of the Oxford WebQ Online 24-Hour Dietary Questionnaire Using Biomarkers. Am J Epidemiol. 2019;188(10):1858-1867. doi:10.1093/aje/kwz165 31318012 PMC7254925

[21] Perez-Cornago A, Pollard Z, Young H, . Description of the updated nutrition calculation of the Oxford WebQ questionnaire and comparison with the previous version among 207,144 participants in UK Biobank. Eur J Nutr. 2021;60(7):4019-4030. doi:10.1007/s00394-021-02558-4 33956230 PMC8437868

[22] Zamora-Ros R, Achaintre D, Rothwell JA, . Urinary excretions of 34 dietary polyphenols and their associations with lifestyle factors in the EPIC cohort study. Sci Rep. 2016;6:26905. doi:10.1038/srep26905 27273479 PMC4895229

[23] Bhagwat S, Haytowitz DB. USDA Database for the Flavonoid Content of Selected Foods, release 3.2. US Department of Agriculture; 2015. Accessed January 18, 2023. https://www.ars.usda.gov/nutrientdata/flav

[24] Bhagwat S, Haytowitz DB, Wasswa-Kintu S. USDA’s Expanded Flavonoid Database for the Assessment of Dietary Intakes, release 1.1. US Department of Agriculture; 2015. Accessed January 18, 2023. https://www.ars.usda.gov/nutrientdata 10.1017/S0007114515001580 26119062

[25] USDA Database for the Proanthocyanidin Content of Selected Foods, release 2 (2015). US Department of Agriculture; 2015. Accessed January 18, 2023. https://agdatacommons.nal.usda.gov/articles/dataset/USDA_Database_for_the_Proanthocyanidin_Content_of_Selected_Foods_-_2004/25060832

[26] Thompson AS, Tresserra-Rimbau A, Karavasiloglou N, . Association of healthful plant-based diet adherence with risk of mortality and major chronic diseases among adults in the UK. JAMA Netw Open. 2023;6(3):e234714. doi:10.1001/jamanetworkopen.2023.4714 36976560 PMC10051114

[27] Kornblith E, Bahorik A, Boscardin WJ, Xia F, Barnes DE, Yaffe K. Association of race and ethnicity with incidence of dementia among older adults. JAMA. 2022;327(15):1488-1495. doi:10.1001/jama.2022.3550 35438728 PMC9020215

[28] Bano S, Rasheed MA, Jamil F, Ibrahim M, Kanwal S. In silico identification of novel apolipoprotein E4 inhibitor for Alzheimer’s disease therapy. Curr Comput Aided Drug Des. 2019;15(1):97-103. doi:10.2174/1573409914666181008164209 30306878

[29] Barberger-Gateau P, Raffaitin C, Letenneur L, . Dietary patterns and risk of dementia: the Three-City Cohort Study. Neurology. 2007;69(20):1921-1930. doi:10.1212/01.wnl.0000278116.37320.52 17998483

[30] Dai Q, Borenstein AR, Wu Y, Jackson JC, Larson EB. Fruit and vegetable juices and Alzheimer’s disease: the Kame Project. Am J Med. 2006;119(9):751-759. doi:10.1016/j.amjmed.2006.03.045 16945610 PMC2266591

[31] Bellou E, Baker E, Leonenko G, ; Alzheimer’s Disease Neuroimaging Initiative. Age-dependent effect of APOE and polygenic component on Alzheimer’s disease. Neurobiol Aging. 2020;93:69-77. doi:10.1016/j.neurobiolaging.2020.04.024 32464432 PMC7308803

[32] Pitharouli MC, Hagenaars SP, Glanville KP, . Elevated C-reactive protein in patients with depression, independent of genetic, health, and psychosocial factors: results from the UK Biobank. Am J Psychiatry. 2021;178(6):522-529. doi:10.1176/appi.ajp.2020.20060947 33985349

[33] Cooper CM, Chin Fatt CR, Liu P, . Discovery and replication of cerebral blood flow differences in major depressive disorder. Mol Psychiatry. 2020;25(7):1500-1510. doi:10.1038/s41380-019-0464-7 31388104

[34] Harshfield EL, Pennells L, Schwartz JE, ; Emerging Risk Factors Collaboration. Association between depressive symptoms and incident cardiovascular diseases. JAMA. 2020;324(23):2396-2405. doi:10.1001/jama.2020.23068 33320224 PMC7739139

[35] Fang JL, Luo Y, Jin SH, Yuan K, Guo Y. Ameliorative effect of anthocyanin on depression mice by increasing monoamine neurotransmitter and up-regulating BDNF expression. J Funct Foods. 2020;66:103757. doi:10.1016/j.jff.2019.103757

[36] Rai A, Gill M, Kinra M, . Catechin ameliorates depressive symptoms in Sprague Dawley rats subjected to chronic unpredictable mild stress by decreasing oxidative stress. Biomed Rep. 2019;11(2):79-84. doi:10.3892/br.2019.1226 31338194 PMC6610213

[37] Gea A, Beunza JJ, Estruch R, ; PREDIMED Group. Alcohol intake, wine consumption and the development of depression: the PREDIMED study. BMC Med. 2013;11:192. doi:10.1186/1741-7015-11-192 23988010 PMC3765610

[38] Schaefer SM, Kaiser A, Behrendt I, Eichner G, Fasshauer M. Association of alcohol types, coffee, and tea intake with risk of dementia: prospective cohort study of UK Biobank participants. Brain Sci. 2022;12(3):360. doi:10.3390/brainsci12030360 35326316 PMC8946788

[39] Rehm J, Hasan OSM, Black SE, Shield KD, Schwarzinger M. Alcohol use and dementia: a systematic scoping review. Alzheimers Res Ther. 2019;11(1):1. doi:10.1186/s13195-018-0453-0 30611304 PMC6320619

[40] Andrews SJ, Goate A, Anstey KJ. Association between alcohol consumption and Alzheimer’s disease: a Mendelian randomization study. Alzheimers Dement. 2020;16(2):345-353. doi:10.1016/j.jalz.2019.09.086 31786126 PMC7057166

[41] National Institute for Health and Care Excellence. Dementia, Disability and Frailty in later Life-Mid-Life Approaches to Delay or Prevent Onset. National Institute for Health and Care Excellence; 2015.

[42] Subar AF, Freedman LS, Tooze JA, . Addressing current criticism regarding the value of self-report dietary data. J Nutr. 2015;145(12):2639-2645. doi:10.3945/jn.115.219634 26468491 PMC4656907

[43] Fry A, Littlejohns TJ, Sudlow C, . Comparison of sociodemographic and health-related characteristics of UK Biobank participants with those of the general population. Am J Epidemiol. 2017;186(9):1026-1034. doi:10.1093/aje/kwx246 28641372 PMC5860371

[44] Matthews FE, Chatfield M, Freeman C, McCracken C, Brayne C; MRC CFAS. Attrition and bias in the MRC Cognitive Function and Ageing Study: an epidemiological investigation. BMC Public Health. 2004;4:12. doi:10.1186/1471-2458-4-12 15113437 PMC419705

[45] Wilkinson T, Schnier C, Bush K, ; Dementias Platform UK and UK Biobank. Identifying dementia outcomes in UK Biobank: a validation study of primary care, hospital admissions and mortality data. Eur J Epidemiol. 2019;34(6):557-565. doi:10.1007/s10654-019-00499-1 30806901 PMC6497624

