# Supplementary material for: Flavonoid-Rich Foods, Dementia Risk, and Interactions With Genetic Risk, Hypertension, and Depression
Source: JAMA Netw Open. 2024 Sep 18;7(9):e2434136. doi: 10.1001/jamanetworkopen.2024.34136 (PMC11411383; doi:10.1001/jamanetworkopen.2024.34136)
Supplement: Supplement 1. — eMethods 1. Flavonoid Subclasses eMethods 2. Healthy Plant-Based Diet Index eMethods 3. Genetic Risk eMethods 4. Covariate Assessment eFigure 1. Flowchart of Participants in the UK Biobank Study eFigure 2. Age- and Sex-Adjusted Kaplan-Meier Survival Curves in All Participants and Stratified by Subgroup in 121 986 Males and Females From the UK Biobank eFigure 3. Risk of Dementia by the Number of Components Met of Tea Intake of ≥5 Servings/d, Red Wine Intake ≥1 Serving/d, and Berry Intake ≥0.5 Servings/d in 121 986 Males and Females From the UK Biobank eTable 1. International Classification of Diseases Codes Used for Ascertainment of Dementia eTable 2. Percent Contribution of Flavonoid-Rich Foods and the Flavodiet Score to Intake of Flavonoid Subclasses in 121 986 Males and Females From the UK Biobank eTable 3. Baseline Characteristics by Inclusion Status in 502 411 Males and Females From the UK Biobank eTable 4. Baseline Characteristics and Dietary Intakes by Dementia Diagnosis in 121 986 Males and Females From the UK Biobank eTable 5. Estimated Hazard Ratios for LASSO Cox Regression Between Risk of Dementia and Flavodiet Score in 121 986 Males and Females From the UK Biobank eTable 6. Risk of Dementia by Quintiles of Flavodiet Score After Removing Each Food From the Final Score in 121 986 Males and Females From the UK Biobank eTable 7. Risk of Dementia by Quintiles of Flavodiet Score and Flavonoid Subclass Intake in 121 986 Males and Females From the UK Biobank, Stratified by Genetic Risk eTable 8. Risk of Dementia by Quintiles of Flavodiet Score and Flavonoid Subclass Intake in 121 105 Males and Females From the UK Biobank, Stratified by Depression Status eTable 9. Risk of Dementia by Quintiles of Flavodiet Score and Flavonoid Subclass Intake in 121 981 Males and Females From the UK Biobank, Stratified by Hypertension Status eTable 10. Risk of Dementia by Quintiles of Flavodiet Score and Flavonoid Subclass Intake in 61 719 Males and Females Aged More Than 60 Years [file jamanetwopen-e2434136-s001.pdf]

Supplemental Online Content

Jennings A, Thompson AS, Tresserra-Rimbau A, et al. Flavonoid-rich foods, dementia risk, and interactions with genetic risk, hypertension, and depression. *JAMA Netw Open*. 2024;7(9):e2434136. doi:10.1001/jamanetworkopen.2024.34136

eMethods 1. Flavonoid Subclasses

eMethods 2. Healthy Plant-Based Diet Index

eMethods 3. Genetic Risk

eMethods 4. Covariate Assessment

eFigure 1. Flowchart of Participants in the UK Biobank Study

eFigure 2. Age- and Sex-Adjusted Kaplan-Meier Survival Curves in All Participants and Stratified by Subgroup in 121 986 Males and Females From the UK Biobank

eFigure 3. Risk of Dementia by the Number of Components Met of Tea Intake of ≥5 Servings/d, Red Wine Intake ≥1 Serving/d, and Berry Intake ≥0.5 Servings/d in 121 986 Males and Females From the UK Biobank

eTable 1. *International Classification of Diseases* Codes Used for Ascertainment of Dementia

eTable 2. Percent Contribution of Flavonoid-Rich Foods and the Flavodiet Score to Intake of Flavonoid Subclasses in 121 986 Males and Females From the UK Biobank

eTable 3. Baseline Characteristics by Inclusion Status in 502 411 Males and Females From the UK Biobank

eTable 4. Baseline Characteristics and Dietary Intakes by Dementia Diagnosis in 121 986 Males and Females From the UK Biobank

eTable 5. Estimated Hazard Ratios for LASSO Cox Regression Between Risk of Dementia and Flavodiet Score in 121 986 Males and Females From the UK Biobank

eTable 6. Risk of Dementia by Quintiles of Flavodiet Score After Removing Each Food From the Final Score in 121 986 Males and Females From the UK Biobank

eTable 7. Risk of Dementia by Quintiles of Flavodiet Score and Flavonoid Subclass Intake in 121 986 Males and Females From the UK Biobank, Stratified by Genetic Risk

eTable 8. Risk of Dementia by Quintiles of Flavodiet Score and Flavonoid Subclass Intake in 121 105 Males and Females From the UK Biobank, Stratified by Depression Status

eTable 9. Risk of Dementia by Quintiles of Flavodiet Score and Flavonoid Subclass Intake in 121 981 Males and Females From the UK Biobank, Stratified by Hypertension Status

eTable 10. Risk of Dementia by Quintiles of Flavodiet Score and Flavonoid Subclass Intake in 61 719 Males and Females Aged More Than 60 Years From the UK Biobank

eTable 11. Risk of Dementia by Quintiles of Flavodiet Score and Flavonoid Subclass Intake in 120 959 Males and Females With at Least 5 Years Follow-Up From the UK Biobank

eTable 12. Risk of Dementia by Quintiles of Flavodiet Score and Flavonoid Subclass Intake in 119 053 Males and Females With No Reported Stroke History From the UK Biobank

eTable 13. Risk of Dementia by Quintiles of Flavodiet Score and Flavonoid Subclass Intake in 35 110 Males and Females At High Genetic Risk of Dementia Whose Reported Ethnicity Was White From the UK Biobank

© 2024 Jennings A et al. *JAMA Network Open*.

eTable 14. Risk of Dementia by Quintiles of Flavodiet Score and Flavonoid Subclass Intake in 121 986 Males and Females From the UK Biobank Adjusted for Individual Foods Associated With Dementia Risk)

eTable 15. Risk of Dementia by Quintiles of Flavodiet Score and Flavonoid Subclass Intake in 53 101 Males and Females From the UK Biobank Living in High Areas of Deprivation or With Low Levels of Education

eTable 16. Risk of Dementia by Quintiles of Flavodiet Score and Flavonoid Subclass Intake in 73 200 Males and Females From the UK Biobank With Low or Moderate Levels of Physical Activity

eReferences

This supplemental material has been provided by the authors to give readers additional information about their work.

© 2024 Jennings A et al. *JAMA Network Open*.

eMethods 1. Flavonoid Subclasses

Intakes were derived for the main subclasses of flavonoids habitually consumed: flavanones (eriodictyol, hesperetin, and naringenin), anthocyanins (cyanidin, delphinidin, malvidin, pelargonidin, petunidin, and peonidin), flavan-3-ols (catechins and epicatechins), flavonols (quercetin, kaempferol, myricetin, and isohamnetin), flavones (luteolin and apigenin), polymers (including proanthocyanidins [excluding monomers], theaflavins, and thearubigins), and proanthocyanidins separately (dimers, trimers, 4–6mers, 7–10mers, polymers, and monomers). Total flavonoid intakes were calculated by summing the 6 component subclasses (flavanones, anthocyanins, flavan-3-ols, flavonols, flavones, and polymers).

© 2024 Jennings A et al. *JAMA Network Open*.

eMethods 2. Healthy Plant-Based Diet Index

The healthy plant-based diet index (hPDI), made up of 17 food groups: whole grains, fruits, vegetables, nuts, legumes and vegetarian protein alternatives, tea and coffee, fruit juices, refined grains, potatoes, sugar-sweetened beverages, sweets and desserts, animal fat, dairy, eggs, fish or seafood, meat, and miscellaneous animal-derived foods, was derived. as previously described [1]

© 2024 Jennings A et al. *JAMA Network Open*.

eMethods 3. Genetic Risk

Genotyping was assayed using the UK BiLEVE Axiom (Affymetrix) and the UK Biobank Axiom arrays (Applied Biosystems)[2]. Imputed genotypes were derived from the Haplotype Reference Consortium and UK10K haplotype resource using computationally efficient methods. The APOE single-nucleotide polymorphisms were directly genotyped by rs429358 and rs7412. A polygenic risk score (PRS) was constructed to capture an individual's load of common genetic variants associated with Alzheimer's disease-related genetic risk in UK Biobank; full details of the methods and the selected SNPs have previously been published [3]. This polygenic risk score was categorized into low (lowest quintile), intermediate (quintiles 2 to 4), and high (highest quintile) risk groups, a routinely used approach for modelling polygenic risk scores in UK Biobank studies [4].

© 2024 Jennings A et al. *JAMA Network Open*.

eMethods 4. Covariate Assessment

Participants completed a touchscreen questionnaire and a verbal interview conducted by trained staff and provided physical measurements and biological samples at baseline. At these assessments data were collected on age, sex, ethnicity, socioeconomic status, education, smoking status, sleep duration, physical activity, BMI, family history of dementia, post-menopausal status, number of self-reported medications and number of long-term health conditions.

History of stroke was defined for participants with a primary or secondary diagnosis of stroke, identified from hospital records or death registries using ICD codes I60, I61, I63 and I64, prior to diagnosis of dementia or end of follow-up. Current depressive symptoms at baseline were measured based on the frequency of four items from the Patient Health Questionnaire: 1) depressed mood, 2) disinterest or absence of enthusiasm, 3) tenseness or restlessness, and 4) tiredness or lethargy, with one point awarded if participants reported symptoms in the previous two weeks. The subsequent depressive symptoms score ranged from 0 to 4 for each participant with depression defined for scores >2 [5].

Systolic blood pressure and diastolic blood pressure were measured twice by using a digital sphygmomanometer (Omron 705 IT; OMRON Healthcare Europe B.V.) with the participants at rest in the seated position for at least five minutes and a one-minute interval between measurements. Hypertension was defined if systolic blood pressure was >140 and/ or diastolic blood pressure >90 and/ or self-reported use of blood pressure medication. For participants, without these data (n=6874) we used a self-reported diagnosis by a medical professional.

© 2024 Jennings A et al. *JAMA Network Open*.

eFigure1. Flowchart of Participants in the UK Biobank Study

[1] Incidence rate per 1000 person-years

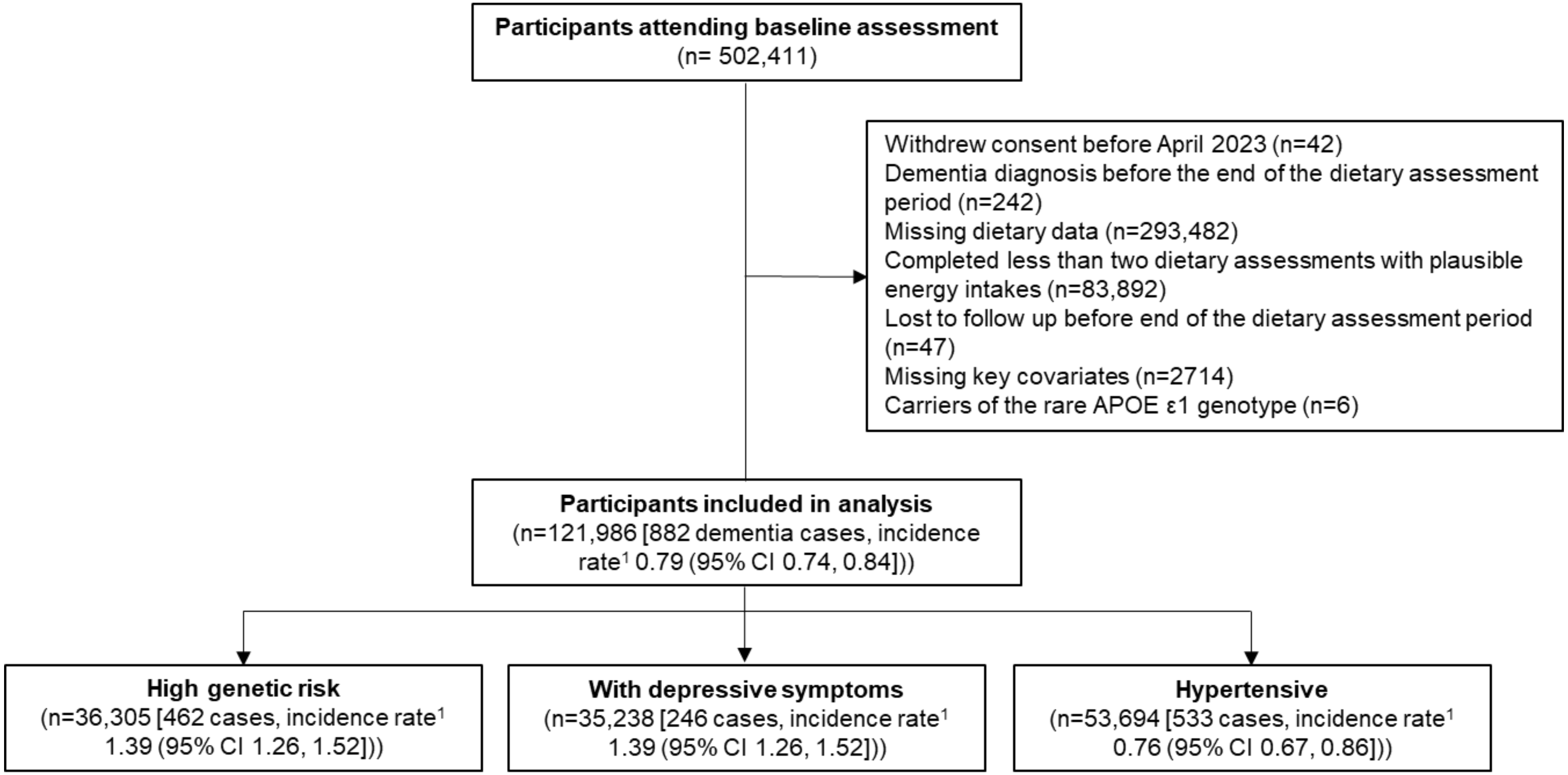

© 2024 Jennings A et al. *JAMA Network Open*.

eFigure2: Age- and Sex-Adjusted Kaplan-Meier Survival Curves in All Participants and Stratified by Subgroup in 121 986 Males and Females From the UK Biobank

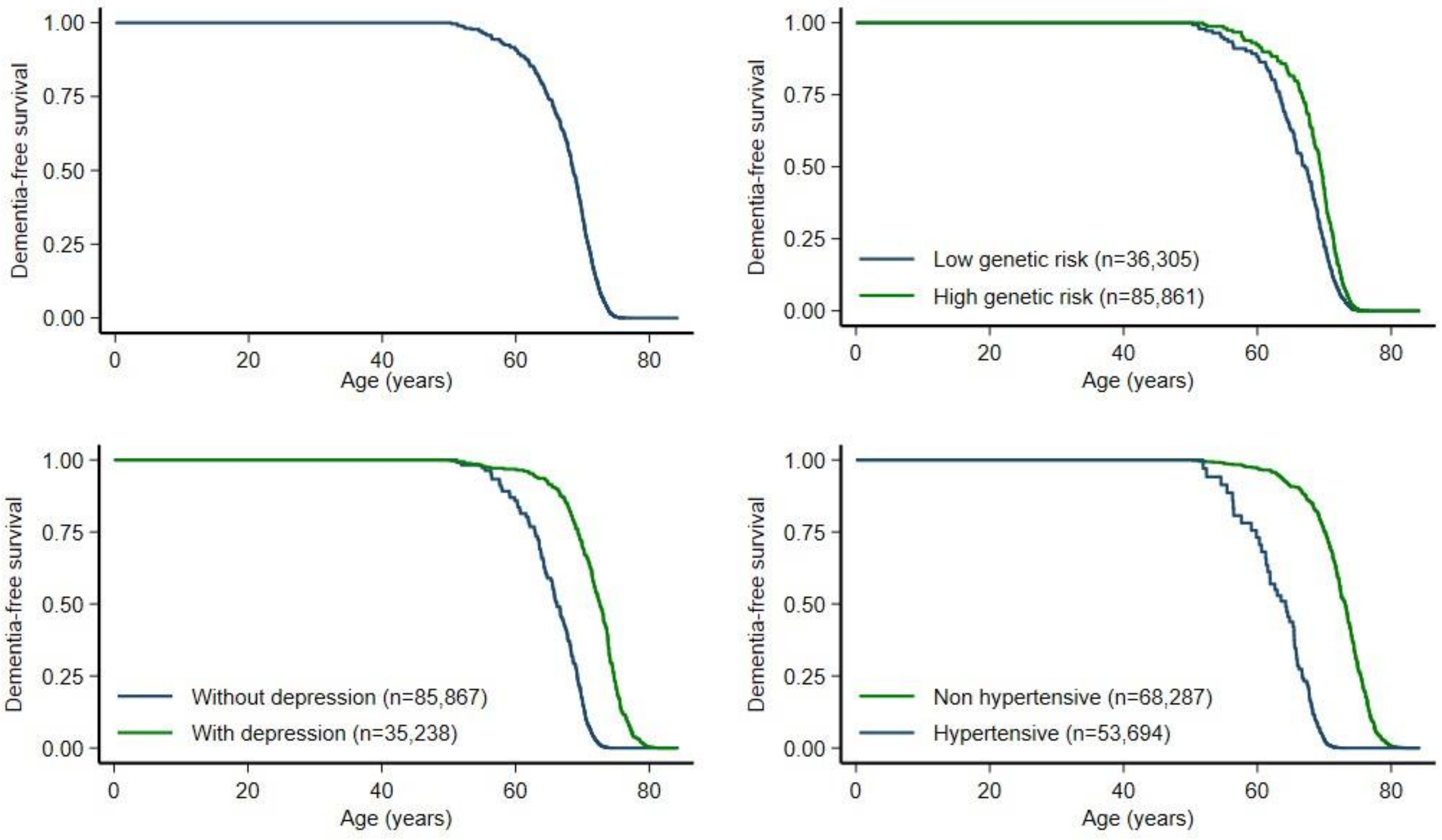

© 2024 Jennings A et al. *JAMA Network Open*.

eFigure 3. Risk of Dementia by the Number of Components Met of Tea Intake of ≥5 Servings/d, Red Wine Intake ≥1 Serving/d, and Berry Intake ≥0.5 Servings/d in 121 986 Males and Females From the UK Biobank

Values are adjusted hazard ratios (95% CI), n=121,986 (882 cases). Foods were identified as those in the flavodiet score significantly associated with reduced dementia risk. The number of ideal servings were defined from the median intake of tea, red wine, and berries in those participants in the highest quintile of all three foods (n=1297). Model adjusted for sex (male or female), socioeconomic status (Townsend Index, categorised as low [quintile 1], moderate [quintiles 2–4], high [quintile 5] deprivation), highest level of education (higher, vocational, upper Secondary, lower Secondary, none or unknown), ethnicity (White, Mixed, Asian, Black, Chinese, other group, not answered), current smoking status (yes or no), typical sleep duration (≤ 6 hours, 7–8 hours or > 8 hours, not answered), physical activity (total excess metabolic equivalents, in quintiles), BMI (kg/m2), family history of dementia (yes or no), history of stroke (yes or no), post-menopausal status (yes, no, male or unknown), number of medications taken (1-3, 4-6, 7-9 or 10+), number of long-term health conditions (1-2, 3, 4, 5+), number of dietary assessments completed with plausible energy intakes (2, 3, 4 or 5), healthy plant-based diet index (score, in quintiles), alcohol intake not from red wine (g/d, in quintiles), energy intake (kcal/d, in quintiles) and fat intake (g/d, in quintiles) and stratified by region (London, North-West England, North-East England, Yorkshire, West Midlands, East Midlands, South-East England, South-West England, Scotland and Wales).

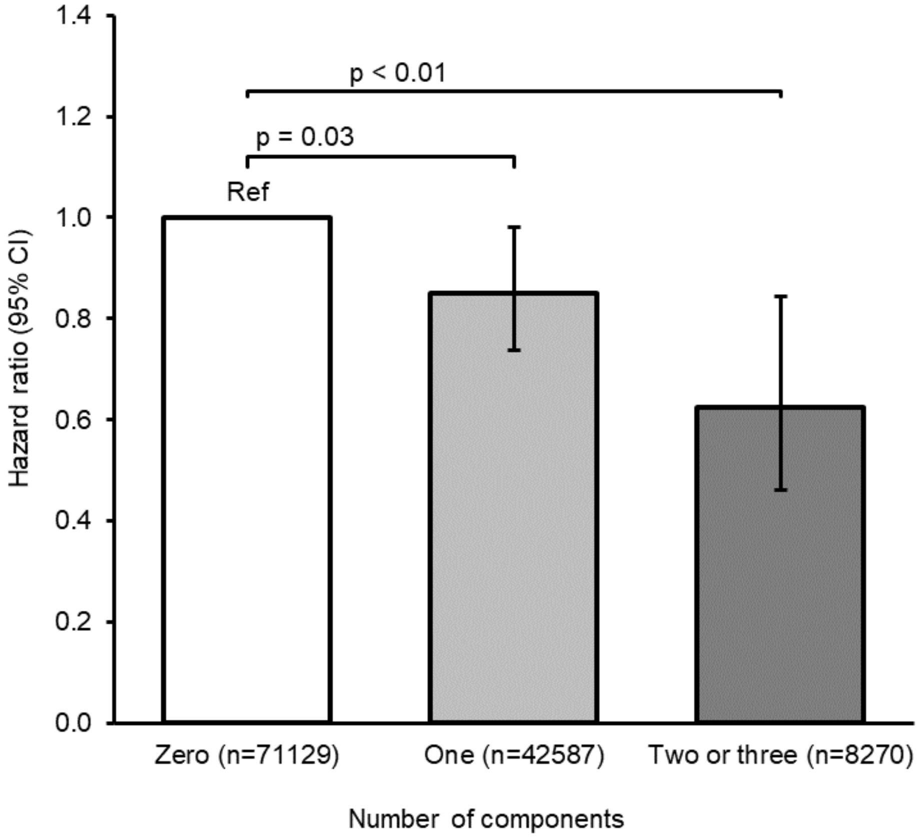

© 2024 Jennings A et al. *JAMA Network Open*.

eTable1. ***International Classification of Diseases*** **Codes Used for Ascertainment of Dementia**

**Codes**	
ICD-9	290.2, 290.3, 290.4, 291.2, 294.1, 331.0, 331.1, 331.2, 331.5
ICD-10	A81.0, F00, F00.0, F00.1, F00.2, F00.9, F01, F01.0, F01.1, F01.2, F01.3, F01.8, F01.9, F02, F02.0, F02.1, F02.2, F02.3, F02.4, F02.8, F03, F05.1, F10.6, G30, G30.0, G30.1, G30.8, G30.9, G31.0, G31.1, G31.8, I67.3

© 2024 Jennings A et al. *JAMA Network Open*.

eTable 2. Percent Contribution of Flavonoid-Rich Foods and the Flavodiet Score to Intake of Flavonoid Subclasses in 121 986 Males and Females From the UK Biobank

Food	Servings/d	Percentage contribution to flavonoid subclass intake							
	Mean (SD)	Flavanone	Anthocyanin	Flavan-3-ol	Flavonol	Flavone	Polymer	Proanthocyanidin[2]	Total Flavonoid
Tea	2.7 (1.8)	0.0	0.0	84.1	54.4	6.9	71.0	48.1	68.7
Apples	0.36 (0.65)	0.0	2.4	2.2	5.4	4.9	7.5	12.5	5.8
Red wine	0.38 (0.48)	6.0	40.4	3.6	2.8	9.5	3.3	6.7	4.7
Grapes	0.17 (0.34)	0.0	14.8	0.4	0.6	10.7	1.9	3.1	1.9
Berries	0.03 (0.10)	0.0	24.3	0.5	1.1	0.4	1.3	2.3	1.9
Dark chocolate	0.19 (0.36)	0.0	0.0	0.4	0.1	0.0	1.7	2.8	1.3
Oranges	0.15 (0.22)	37.6	0.0	0.0	0.3	2.6	0.0	0.0	1.1
Onions	0.34 (0.51)	0.0	0.0	0.0	7.2	0.2	0.0	0.0	0.3
Grapefruit	0.09 (0.17)	3.7	0.0	0.0	0.0	1.3	0.0	0.0	0.1
Peppers	0.02 (0.10)	0.0	0.0	0.0	0.3	17.2	0.0	0.0	0.0
flavodiet score[1]	4.4 (2.2)	47.4	82.0	91.1	72.4	53.6	86.7	75.4	85.8

[1] flavodiet score was calculated by summing intakes (in servings per day) of tea (black and green), red wine, apples, berries, grapes, oranges, grapefruit, sweet peppers, onions, and dark chocolate.
[2]Proanthocyanidins are also included in the polymer subclass.

© 2024 Jennings A et al. *JAMA Network Open*.

eTable 3. Baseline Characteristics by Inclusion Status in 502 411 Males and Females From the UK Biobank.

Characteristic	Included sample (n=121986)	Excluded sample (n=380425)	Data available (n=)
Age (y)	57.0 [50.0, 62.0]	58.0 [50.0, 63.0]	502408
Physical activity (excess METs)	1176 [512, 2334]	1171 [456, 2586]	482032
BMI (kg/m2)	26.0 [23.6, 29.0]	27.0 [24.4, 30.2]	499304
Sex			
Female	67866 (55.6%)	205459 (54.0%)	
Male	54120 (44.4%)	174966 (46.0%)	502411
Ethnicity			
Asian	1115 (0.9%)	8764 (2.3%)	
Black	930 (0.8%)	7128 (1.9%)	
Chinese	310 (0.3%)	1263 (0.3%)	
Mixed	651 (0.5%)	2303 (0.6%)	
White	117918 (96.7%)	354694 (93.2%)	
Other	698 (0.6%)	3859 (1.0%)	
Unknown	364 (0.3%)	2414 (0.6%)	502411
Townsend Deprivation			
Low deprivation	26757 (21.9%)	73633 (19.4%)	
Moderate deprivation	75746 (62.1%)	225294 (59.3%)	
High deprivation	19483 (16.0%)	80872 (21.3%)	501785
Education level			
None	7731 (6.3%)	77528 (20.4%)	
Lower secondary	27924 (22.9%)	104137 (27.4%)	
Upper secondary	16418 (13.5%)	38890 (10.2%)	
Vocational	5945 (4.9%)	26779 (7.0%)	
Higher	63653 (52.2%)	123274 (32.4%)	
Unknown	315 (0.3%)	9817 (2.6%)	502411
Current smoker			
No	113604 (93.1%)	334524 (88.2%)	
Yes	8382 (6.9%)	44580 (11.8%)	501090
Sleep duration			
<=6 hours	26218 (21.5%)	97010 (25.5%)	
7-8 hours	87991 (72.1%)	248635 (65.4%)	
> 8 hours	7575 (6.2%)	31655 (8.3%)	
Unknown	202 (0.2%)	3125 (0.8%)	502411
Family history of dementia			
No	105681 (86.6%)	338310 (88.9%)	
Yes	16305 (13.4%)	42115 (11.1%)	502411
Stroke history			
No	119053 (97.6%)	365468 (96.1%)	
Yes	2933 (2.4%)	14957 (3.9%)	502411
Post menopausal			
No	17612 (14.4%)	46431 (12.2%)	
Yes	40032 (32.8%)	125348 (33.0%)	
Male	54120 (44.4%)	174966 (46.0%)	
Unknown	10222 (8.4%)	33204 (8.7%)	501935
Number of medications taken			
None	37977 (31.1%)	99654 (26.2%)	
1-3	56923 (46.7%)	173242 (45.5%)	
4-6	19997 (16.4%)	72616 (19.1%)	
7-9	5232 (4.3%)	23966 (6.3%)	
>10	1857 (1.5%)	10947 (2.9%)	502411
Number long term conditions			
0	43178 (35.4%)	125315 (32.9%)	

© 2024 Jennings A et al. *JAMA Network Open*.

1	39841 (32.7%)	122513 (32.2%)	
2	22568 (18.5%)	74356 (19.5%)	
>3	16399 (13.4%)	58241 (15.3%)	502411
APOE ε4 carrier			
No	86086 (72.1%)	262016 (71.4%)	
Yes	33317 (27.9%)	104946 (28.6%)	486365
Genetic risk category			
Low risk	24100 (20.2%)	73124 (19.9%)	
Medium risk	71830 (60.2%)	219841 (59.9%)	
High risk	23424 (19.6%)	73799 (20.1%)	486118
Genetic kinship			
At least one relative identified	33365 (27.4%)	114312 (30.0%)	
No kinship found	86191 (70.7%)	253325 (66.6%)	
Unknown	2430 (2.0%)	12788 (3.4%)	502411
Depressive symptoms			
No	65 (0.1%)	820 (0.2%)	
Yes	121921 (99.9%)	378730 (99.8%)	501536
Hypertension			
No	68287 (56.0%)	197426 (51.9%)	
Yes	53694 (44.0%)	182878 (48.1%)	502285

Values are median [IQR] or n= (%).

© 2024 Jennings A et al. *JAMA Network Open*.

eTable 4. Baseline Characteristics and Dietary Intakes by Dementia Diagnosis in 121 986 Males and Females From the UK Biobank

Characteristic	All (n=121986)	No dementia (n=121104)	Incident dementia (n=882)
Age (y)	57.0 [50.0, 62.0]	57.0 [50.0, 62.0]	65.0 [62.0, 67.0]
Duration of follow up (y)	9.4 [9.3, 9.8]	9.4 [9.3, 9.8]	7.1 [5.5, 8.4]
Physical activity (excess METs)	1176 [512, 2334]	1176 [512, 2334]	1065 [525, 2349]
BMI (kg/m2)	26.0 [23.6, 29.0]	26.0 [23.6, 29.0]	26.2 [23.7, 29.5]
Flavanones (mg/d)	17.9 [5.2, 35.7]	17.9 [5.2, 35.7]	19.1 [5.2, 37.8]
Anthocyanins (mg/d)	20.5 [7.1, 40.3]	20.5 [7.1, 40.3]	20.7 [7.2, 41.1]
Flavan-3-ols (mg/d)	166 [85.1, 247]	166 [85.2, 247]	161 [78.1, 235]
Flavonols (mg/d)	31.8 [20.3, 43.5]	31.8 [20.3, 43.5]	31.9 [19.1, 43.5]
Flavones (mg/d)	0.86 [0.46, 1.4]	0.86 [0.46, 1.4]	0.85 [0.45, 1.4]
Polymers (mg/d)	520 [268, 767]	520 [268, 767]	533 [273, 761]
Proanthocyanidins (mg/d)	336 [230, 446]	336 [230, 446]	344 [232, 448]
Total Flavonoids (mg/d)	793 [452, 1119]	793 [452, 1119]	808 [444, 1126]
flavodiet score	4.3 [2.8, 5.9]	4.3 [2.8, 5.9]	4.3 [2.8, 5.8]
flavodiet score (tea capped at 4 cups)	4.2 [2.8, 5.3]	4.2 [2.8, 5.3]	4.3 [2.8, 5.4]
Tea (servings/d)	2.7 [1.0, 4.0]	2.7 [1.2, 4.0]	2.5 [1.0, 3.8]
Red wine (servings/d)	0.00 [0.00, 0.50]	0.00 [0.00, 0.50]	0.00 [0.00, 0.50]
Apples (servings/d)	0.25 [0.00, 0.67]	0.25 [0.00, 0.67]	0.33 [0.00, 0.75]
Grapes (servings/d)	0.00 [0.00, 0.25]	0.00 [0.00, 0.25]	0.00 [0.00, 0.25]
Dark chocolate (servings/d)	0.00 [0.00, 0.00]	0.00 [0.00, 0.00]	0.00 [0.00, 0.00]
Berries (servings/d)	0.00 [0.00, 0.25]	0.00 [0.00, 0.25]	0.00 [0.00, 0.25]
Onions (servings/d)	0.05 [0.00, 0.25]	0.05 [0.00, 0.25]	0.00 [0.00, 0.17]
Oranges (servings/d)	0.00 [0.00, 0.50]	0.00 [0.00, 0.50]	0.00 [0.00, 0.67]
Peppers (servings/d)	0.00 [0.00, 0.13]	0.00 [0.00, 0.13]	0.00 [0.00, 0.08]
Grapefruit (servings/d)	0.00 [0.00, 0.00]	0.00 [0.00, 0.00]	0.00 [0.00, 0.00]
Energy (kcal/d)	2009 [1718, 2332]	2009 [1718, 2332]	2013 [1719, 2360]
Fat (g/d)	70.4 [56.1, 86.5]	70.4 [56.1, 86.5]	71.2 [55.3, 87.1]
Alcohol not from red wine (g)	4.6 [0.00, 16.5]	4.7 [0.00, 16.5]	4.3 [0.00, 14.6]
hPDI (score)	54.0 [49.0, 58.0]	54.0 [49.0, 58.0]	54.0 [50.0, 59.0]
Days of dietary assessment completed	3.0 [2.0, 4.0]	3.0 [2.0, 4.0]	3.0 [2.0, 3.0]
Sex			
Female	67866 (55.6%)	67447 (55.7%)	419 (47.5%)
Male	54120 (44.4%)	53657 (44.3%)	463 (52.5%)
Ethnicity			
Asian	1115 (0.9%)	1112 (0.9%)	3 (0.3%)
Black	930 (0.8%)	927 (0.8%)	3 (0.3%)
Chinese	310 (0.3%)	309 (0.3%)	1 (0.1%)
Mixed	651 (0.5%)	649 (0.5%)	2 (0.2%)
White	117918 (96.7%)	117051 (96.7%)	867 (98.3%)
Other	698 (0.6%)	695 (0.6%)	3 (0.3%)
Unknown	364 (0.3%)	361 (0.3%)	3 (0.3%)
Townsend Deprivation			
Low deprivation	24435 (20.0%)	24246 (20.0%)	189 (21.4%)
Moderate deprivation	73154 (60.0%)	72644 (60.0%)	510 (57.8%)
High deprivation	24397 (20.0%)	24214 (20.0%)	183 (20.7%)
Education level			
None	7731 (6.3%)	7612 (6.3%)	119 (13.5%)
Lower secondary	27924 (22.9%)	27726 (22.9%)	198 (22.4%)
Upper secondary	16418 (13.5%)	16314 (13.5%)	104 (11.8%)
Vocational	5945 (4.9%)	5894 (4.9%)	51 (5.8%)
Higher	63653 (52.2%)	63245 (52.2%)	408 (46.3%)
Unknown	315 (0.3%)	313 (0.3%)	2 (0.2%)
Current smoker			

© 2024 Jennings A et al. *JAMA Network Open*.

No	113604 (93.1%)	112786 (93.1%)	818 (92.7%)
Yes	8382 (6.9%)	8318 (6.9%)	64 (7.3%)
Sleep duration			
<=6 hours	26218 (21.5%)	26016 (21.5%)	202 (22.9%)
7-8 hours	87991 (72.1%)	87398 (72.2%)	593 (67.2%)
> 8 hours	7575 (6.2%)	7489 (6.2%)	86 (9.8%)
Unknown	202 (0.2%)	201 (0.2%)	1 (0.1%)
Family history of dementia			
No	105681 (86.6%)	105042 (86.7%)	639 (72.4%)
Yes	16305 (13.4%)	16062 (13.3%)	243 (27.6%)
Stroke history			
No	119053 (97.6%)	118265 (97.7%)	788 (89.3%)
Yes	2933 (2.4%)	2839 (2.3%)	94 (10.7%)
Post menopausal			
No	17612 (14.4%)	17595 (14.5%)	17 (1.9%)
Yes	40032 (32.8%)	39696 (32.8%)	336 (38.1%)
Male	54120 (44.4%)	53657 (44.3%)	463 (52.5%)
Unknown	10222 (8.4%)	10156 (8.4%)	66 (7.5%)
Number of medications taken			
None	37977 (31.1%)	37830 (31.2%)	147 (16.7%)
1-3	56923 (46.7%)	56578 (46.7%)	345 (39.1%)
4-6	19997 (16.4%)	19755 (16.3%)	242 (27.4%)
7-9	5232 (4.3%)	5145 (4.2%)	87 (9.9%)
>10	1857 (1.5%)	1796 (1.5%)	61 (6.9%)
Number long term conditions			
0	43178 (35.4%)	42996 (35.5%)	182 (20.6%)
1	39841 (32.7%)	39583 (32.7%)	258 (29.3%)
2	22568 (18.5%)	22350 (18.5%)	218 (24.7%)
>3	16399 (13.4%)	16175 (13.4%)	224 (25.4%)
APOE ε4 carrier			
No	86086 (72.1%)	85662 (72.3%)	424 (49.0%)
Yes	33317 (27.9%)	32876 (27.7%)	441 (51.0%)
Genetic risk category			
Low risk	23872 (20.0%)	23783 (20.1%)	89 (10.3%)
Medium risk	71612 (60.0%)	71190 (60.1%)	422 (48.8%)
High risk	23870 (20.0%)	23517 (19.8%)	353 (40.9%)
Genetic kinship			
At least one relative identified	33365 (27.4%)	33104 (27.3%)	261 (29.6%)
No kinship found	86191 (70.7%)	85586 (70.7%)	605 (68.6%)
Unknown	2430 (2.0%)	2414 (2.0%)	16 (1.8%)
Depressive symptoms			
No	65 (0.1%)	65 (0.1%)	0 (0.0%)
Yes	121921 (99.9%)	121039 (99.9%)	882 (100.0%)
Hypertension			
No	68287 (56.0%)	67938 (56.1%)	349 (39.6%)
Yes	53694 (44.0%)	53161 (43.9%)	533 (60.4%)

Values are median [IQR] or n= (%). [1]Proanthocyanidins are also included in the polymer subclass. [2]flavodiet score was calculated by summing intakes (in servings per day) of tea (black and green), red wine, apples, berries, grapes, oranges, grapefruit, sweet peppers, onions, and dark chocolate.

© 2024 Jennings A et al. *JAMA Network Open*.

eTable 5. Estimated Hazard Ratios for LASSO Cox Regression Between Risk of Dementia and Flavodiet Score in 121 986 Males and Females From the UK Biobank

Variable	Coeffcients[1]
flavodiet score (quintiles)[2]	0.95
Sex	1.12
Townsend Index	1.08
Highest level of education	0.98
Ethnicity	0
Current smoking status	1.46
Typical sleep duration	0
Physical activity	0.98
BMI	0
Family history of dementia	2.06
History of stroke	2.53
Post-menopausal status	0
Number of medications taken	1.35
Number of dietary assessments completed	0.87
Healthy plant-based diet index	0
Alcohol intake not from red wine	0.96
Energy intake	1.03
Fat intake	0

[1] Values are the post selection coefficients of the unstandardized variables calculated from LASSO Cox regression. [2]flavodiet score was calculated by summing intakes (in servings per day) of tea (black and green), red wine, apples, berries, grapes, oranges, grapefruit, sweet peppers, onions, and dark chocolate, values relate to per quintile of the score.

© 2024 Jennings A et al. *JAMA Network Open*.

eTable 6. Risk of Dementia by Quintiles of Flavodiet Score After Removing Each Food From the Final Score in 121 986 Males and Females From the UK Biobank

Intake component	Q1	Q2	Q3	Q4	Q5	P-trend
**flavodiet score (tea capped at 4 cups)**						
n= (cases)	23379 (174)	24166 (163)	24589 (170)	24484 (188)	25368 (187)	
HR (95% CI)	1.00	0.79 (0.64, 0.99)	0.79 (0.64, 0.98)	0.81 (0.66, 1.01)	0.74 (0.60, 0.93)	0.03
**flavodiet score (w/o tea)**						
n= (cases)	20777 (124)	26392 (189)	25803 (182)	24339 (188)	24675 (199)	
HR (95% CI)	1.00	1.04 (0.83, 1.31)	0.99 (0.79, 1.25)	1.05 (0.83, 1.33)	1.03 (0.81, 1.32)	0.83
**flavodiet score (w/o red wine)**						
n= (cases)	23902 (176)	24875 (170)	23301 (164)	24285 (188)	25623 (184)	
HR (95% CI)	1.00	0.83 (0.67, 1.02)	0.82 (0.66, 1.02)	0.86 (0.70, 1.07)	0.81 (0.65, 1.01)	0.16
**flavodiet score (w/o apples)**						
n= (cases)	24023 (183)	23137 (171)	25922 (181)	24215 (174)	24689 (173)	
HR (95% CI)	1.00	0.84 (0.68, 1.03)	0.75 (0.61, 0.93)	0.74 (0.59, 0.91)	0.71 (0.57, 0.89)	<0.01
**flavodiet score (w/o grapes)**						
n= (cases)	24225 (183)	24256 (163)	24592 (189)	24127 (174)	24786 (173)	
HR (95% CI)	1.00	0.79 (0.64, 0.97)	0.87 (0.70, 1.07)	0.78 (0.63, 0.97)	0.74 (0.59, 0.93)	0.02
**flavodiet score (w/o dark chocolate)**						
n= (cases)	23441 (176)	23896 (160)	24518 (175)	24553 (193)	25578 (178)	
HR (95% CI)	1.00	0.78 (0.63, 0.97)	0.77 (0.62, 0.96)	0.85 (0.69, 1.06)	0.71 (0.57, 0.89)	0.03
**flavodiet score (w/o berries)**						
n= (cases)	22366 (165)	26237 (179)	23157 (162)	25569 (194)	24657 (182)	
HR (95% CI)	1.00	0.80 (0.65, 0.99)	0.80 (0.65, 1.00)	0.82 (0.66, 1.02)	0.77 (0.61, 0.96)	0.07
**flavodiet score (w/o onions)**						
n= (cases)	24380 (177)	23542 (163)	25130 (175)	24063 (200)	24871 (167)	
HR (95% CI)	1.00	0.82 (0.66, 1.02)	0.79 (0.64, 0.97)	0.91 (0.74, 1.13)	0.71 (0.57, 0.89)	0.03
**flavodiet score (w/o oranges)**						
n= (cases)	23471 (184)	24514 (157)	25080 (204)	24053 (171)	24868 (166)	
HR (95% CI)	1.00	0.71 (0.57, 0.88)	0.85 (0.69, 1.04)	0.72 (0.58, 0.89)	0.67 (0.54, 0.84)	<0.01
**flavodiet score (w/o peppers)**						
n= (cases)	23797 (178)	24081 (157)	24579 (179)	24051 (193)	25478 (175)	
HR (95% CI)	1.00	0.76 (0.61, 0.95)	0.79 (0.64, 0.98)	0.85 (0.69, 1.05)	0.70 (0.56, 0.88)	0.02
**flavodiet score (w/o grapefruit)**						
n= (cases)	23574 (173)	23682 (165)	24524 (172)	24605 (195)	25601 (177)	
HR (95% CI)	1.00	0.83 (0.67, 1.03)	0.78 (0.63, 0.97)	0.88 (0.71, 1.08)	0.73 (0.58, 0.91)	0.03

Values are adjusted hazard ratios (95% CI), n=121,986 (882 cases). flavodiet score was calculated by summing intakes (in servings per day) of tea (black and green), red wine, apples, berries, grapes, oranges, grapefruit, sweet peppers, onions, and dark chocolate. Models adjusted for sex (male or female), socioeconomic status (Townsend Index, categorised as low [quintile 1], moderate [quintiles 2–4], high [quintile 5] deprivation), highest level of education (higher, vocational, upper Secondary, lower Secondary, none or unknown), ethnicity (White, Mixed, Asian, Black, Chinese, other group, not answered), current smoking status (yes or no), typical sleep duration (≤ 6 hours, 7–8 hours or > 8 hours, not answered), physical activity (total excess metabolic equivalents, in quintiles), BMI (kg/m^2), family history of dementia (yes or no), history of stroke (yes or no), post-menopausal status (yes, no, male or unknown), number of medications taken (1-3, 4-6, 7-9 or 10+), number of long-term health conditions (1-2, 3, 4, 5+), number of dietary assessments completed with plausible energy intakes (2, 3, 4 or 5), healthy plant-based diet index (score, in quintiles), alcohol intake not from red wine (g/d, in quintiles), energy intake (kcal/d, in quintiles) and fat intake (g/d, in quintiles) and stratified by region (London, North-West England, North-East England, Yorkshire, West Midlands, East Midlands, South-East England, South-West England, Scotland and Wales).

© 2024 Jennings A et al. *JAMA Network Open*.

eTable 7. Risk of Dementia by Quintiles of Flavodiet Score and Flavonoid Subclass Intake in 121 986 Males and Females From the UK Biobank, Stratified by Genetic Risk

Intake component	Q1	Q2	Q3	Q4	Q5	P-trend	P-interaction
**High genetic risk**							
**flavodiet score (points)**[1]							
n= (cases)	7438 (112)	7559 (85)	6942 (84)	7131 (102)	7235 (79)		
HR (95% CI)	1.00	0.62 (0.46, 0.82)	0.64 (0.48, 0.86)	0.74 (0.56, 0.98)	0.57 (0.42, 0.78)	0.01	0.02
**Flavanones (mg/d)**							
n= (cases)	7262 (91)	7261 (83)	7261 (91)	7261 (89)	7260 (108)		
HR (95% CI)	1.00	0.89 (0.66, 1.20)	0.93 (0.69, 1.25)	0.88 (0.66, 1.19)	1.01 (0.76, 1.35)	0.92	0.77
**Anthocyanins (mg/d)**							
n= (cases)	7262 (95)	7261 (85)	7261 (102)	7261 (97)	7260 (83)		
HR (95% CI)	1.00	0.72 (0.53, 0.97)	0.82 (0.62, 1.10)	0.72 (0.54, 0.97)	0.60 (0.44, 0.82)	<0.01	0.01
**Flavan-3-ols (mg/d)**							
n= (cases)	7262 (112)	7261 (92)	7261 (96)	7261 (84)	7260 (78)		
HR (95% CI)	1.00	0.70 (0.53, 0.93)	0.72 (0.54, 0.95)	0.61 (0.46, 0.82)	0.58 (0.43, 0.79)	<0.01	0.05
**Flavonols (mg/d)**							
n= (cases)	7262 (117)	7261 (94)	7262 (75)	7260 (88)	7260 (88)		
HR (95% CI)	1.00	0.67 (0.51, 0.89)	0.53 (0.39, 0.71)	0.59 (0.44, 0.78)	0.58 (0.43, 0.78)	<0.01	0.03
**Flavones (mg/d)**							
n= (cases)	7262 (94)	7261 (87)	7261 (97)	7261 (97)	7260 (87)		
HR (95% CI)	1.00	0.81 (0.60, 1.08)	0.85 (0.63, 1.14)	0.87 (0.65, 1.17)	0.78 (0.56, 1.07)	0.26	1.00
**Polymers (mg/d)**							
n= (cases)	7262 (96)	7261 (97)	7261 (98)	7261 (92)	7260 (79)		
HR (95% CI)	1.00	0.81 (0.61, 1.07)	0.84 (0.63, 1.12)	0.72 (0.54, 0.97)	0.64 (0.47, 0.88)	0.01	0.07
**Proanthocyanidins (mg/d)** [2]							
n= (cases)	7262 (103)	7261 (96)	7261 (92)	7261 (82)	7260 (89)		
HR (95% CI)	1.00	0.80 (0.61, 1.07)	0.72 (0.54, 0.97)	0.61 (0.45, 0.82)	0.63 (0.46, 0.86)	<0.01	0.07
**Total flavonoids (mg/d)**							
n= (cases)	7262 (112)	7261 (83)	7261 (97)	7261 (89)	7260 (81)		
HR (95% CI)	1.00	0.62 (0.47, 0.83)	0.71 (0.54, 0.94)	0.61 (0.46, 0.81)	0.58 (0.43, 0.78)	<0.01	0.02
**Low genetic risk**							
**flavodiet score (points)**[1]							
n= (cases)	17741 (74)	16634 (78)	17824 (95)	16543 (85)	16939 (88)		
HR (95% CI)	1.00	1.02 (0.74, 1.40)	1.11 (0.81, 1.51)	1.04 (0.75, 1.44)	0.99 (0.71, 1.38)	0.99	-
**Flavanones (mg/d)**							
n= (cases)	17137 (85)	17137 (89)	17135 (74)	17137 (83)	17135 (89)		
HR (95% CI)	1.00	1.01 (0.75, 1.36)	0.83 (0.60, 1.13)	0.88 (0.65, 1.20)	0.92 (0.68, 1.25)	0.40	-
**Anthocyanins (mg/d)**							
n= (cases)	17137 (66)	17137 (88)	17135 (79)	17137 (86)	17135 (101)		
HR (95% CI)	1.00	1.16 (0.84, 1.61)	0.96 (0.69, 1.34)	0.99 (0.71, 1.38)	1.06 (0.77, 1.48)	0.90	-

© 2024 Jennings A et al. *JAMA Network Open*.

**Flavan-3-ols (mg/d)**							
n= (cases)	17137 (80)	17137 (80)	17135 (93)	17137 (79)	17135 (88)		
HR (95% CI)	1.00	0.94 (0.69, 1.28)	1.00 (0.74, 1.35)	0.86 (0.62, 1.18)	0.92 (0.67, 1.25)	0.47	-
**Flavonols (mg/d)**							
n= (cases)	17137 (79)	17137 (77)	17135 (76)	17137 (98)	17135 (90)		
HR (95% CI)	1.00	0.89 (0.65, 1.23)	0.81 (0.59, 1.11)	1.03 (0.76, 1.40)	0.91 (0.66, 1.25)	0.95	-
**Flavones (mg/d)**							
n= (cases)	17137 (87)	17137 (77)	17135 (92)	17137 (90)	17135 (74)		
HR (95% CI)	1.00	0.76 (0.56, 1.04)	0.91 (0.67, 1.23)	0.84 (0.61, 1.14)	0.67 (0.48, 0.94)	0.08	-
**Polymers (mg/d)**							
n= (cases)	17137 (71)	17137 (74)	17135 (98)	17137 (89)	17135 (88)		
HR (95% CI)	1.00	0.95 (0.68, 1.32)	1.18 (0.86, 1.61)	1.04 (0.76, 1.43)	0.99 (0.72, 1.38)	0.86	-
**Proanthocyanidins (mg/d)** [2]							
n= (cases)	17137 (74)	17137 (72)	17135 (81)	17137 (99)	17135 (94)		
HR (95% CI)	1.00	0.87 (0.63, 1.21)	0.92 (0.66, 1.27)	1.06 (0.78, 1.46)	0.97 (0.69, 1.36)	0.67	-
**Total flavonoids (mg/d)**							
n= (cases)	17137 (74)	17137 (77)	17135 (90)	17137 (90)	17135 (89)		
HR (95% CI)	1.00	0.95 (0.69, 1.32)	1.02 (0.74, 1.39)	0.99 (0.72, 1.35)	0.95 (0.69, 1.31)	0.86	-

Values are adjusted hazard ratios (95% CI). Participants at high genetic risk were those in the highest quintile of PRS (n=2988), a carrier of the APOE ε4 genotype (n=12435) or both (n=20882) [1] flavodiet score was calculated by summing intakes (in servings per day) of tea (black and green), red wine, apples, berries, grapes, oranges, grapefruit, sweet peppers, onions, and dark chocolate. [2]Proanthocyanidins are also included in the polymer subclass. Models adjusted for sex (male or female), socioeconomic status (Townsend Index, categorised as low [quintile 1], moderate [quintiles 2–4], high [quintile 5] deprivation), highest level of education (higher, vocational, upper Secondary, lower Secondary, none or unknown), ethnicity (White, Mixed, Asian, Black, Chinese, other group, not answered), current smoking status (yes or no), typical sleep duration (≤ 6 hours, 7–8 hours or > 8 hours, not answered), physical activity (total excess metabolic equivalents, in quintiles), BMI (kg/m^2), family history of dementia (yes or no), history of stroke (yes or no), post-menopausal status (yes, no, male or unknown), number of medications taken (1-3, 4-6, 7-9 or 10+), number of long-term health conditions (1-2, 3, 4, 5+), genetic kinship (at least one relative, no kinship, unknown), number of dietary assessments completed with plausible energy intakes (2, 3, 4 or 5), healthy plant-based diet index (score, in quintiles), alcohol intake not from red wine (g/d, in quintiles), energy intake (kcal/d, in quintiles) and fat intake (g/d, in quintiles) and stratified by region (London, North-West England, North-East England, Yorkshire, West Midlands, East Midlands, South-East England, South-West England, Scotland and Wales). P-interaction was calculated using all participants and refers to the interaction term between intake and indicator of genetic risk (high vs low) in the highest quintile of intake.

© 2024 Jennings A et al. *JAMA Network Open*.

eTable 8. Risk of Dementia by Quintiles of Flavodiet Score and Flavonoid Subclass Intake in 121 105 Males and Females From the UK Biobank, Stratified by Depression Status

Intake component	Q1	Q2	Q3	Q4	Q5	P-trend	P-interaction
**With depression**							
**flavodiet score (points)[1]**							
n= (cases)	7553 (63)	6814 (46)	7074 (47)	6923 (55)	6874 (35)		
HR (95% CI)	1.00	0.73 (0.50, 1.07)	0.66 (0.45, 0.98)	0.78 (0.54, 1.15)	0.52 (0.33, 0.81)	0.01	0.01
**Flavanones (mg/d)**							
n= (cases)	7049 (51)	7047 (54)	7048 (49)	7047 (38)	7047 (54)		
HR (95% CI)	1.00	1.02 (0.69, 1.50)	0.94 (0.63, 1.41)	0.69 (0.45, 1.06)	0.95 (0.64, 1.41)	0.32	0.55
**Anthocyanins (mg/d)**							
n= (cases)	7049 (46)	7047 (38)	7048 (50)	7047 (58)	7047 (54)		
HR (95% CI)	1.00	0.68 (0.44, 1.06)	0.84 (0.55, 1.27)	0.91 (0.60, 1.36)	0.81 (0.54, 1.24)	0.86	0.76
**Flavan-3-ols (mg/d)**							
n= (cases)	7049 (63)	7047 (52)	7048 (47)	7047 (49)	7047 (35)		
HR (95% CI)	1.00	0.78 (0.54, 1.14)	0.62 (0.42, 0.91)	0.65 (0.44, 0.95)	0.49 (0.32, 0.76)	<0.01	<0.01
**Flavonols (mg/d)**							
n= (cases)	7049 (59)	7047 (46)	7048 (47)	7047 (47)	7047 (47)		
HR (95% CI)	1.00	0.68 (0.46, 1.00)	0.64 (0.43, 0.95)	0.65 (0.44, 0.96)	0.64 (0.42, 0.97)	0.05	0.13
**Flavones (mg/d)**							
n= (cases)	7049 (50)	7047 (45)	7048 (60)	7047 (41)	7047 (50)		
HR (95% CI)	1.00	0.82 (0.55, 1.24)	1.03 (0.70, 1.51)	0.73 (0.47, 1.13)	0.93 (0.60, 1.44)	0.64	0.80
**Polymers (mg/d)**							
n= (cases)	7049 (51)	7047 (50)	7048 (61)	7047 (49)	7047 (35)		
HR (95% CI)	1.00	0.89 (0.60, 1.33)	1.03 (0.71, 1.51)	0.74 (0.50, 1.11)	0.57 (0.36, 0.89)	0.01	0.04
**Proanthocyanidins (mg/d) [2]**							
n= (cases)	7049 (55)	7047 (46)	7048 (49)	7047 (54)	7047 (42)		
HR (95% CI)	1.00	0.73 (0.49, 1.09)	0.77 (0.52, 1.14)	0.79 (0.53, 1.18)	0.63 (0.40, 0.98)	0.10	0.03
**Total flavonoids (mg/d)**							
n= (cases)	7049 (55)	7047 (52)	7048 (55)	7047 (44)	7047 (40)		
HR (95% CI)	1.00	0.88 (0.60, 1.30)	0.84 (0.57, 1.23)	0.64 (0.43, 0.96)	0.61 (0.40, 0.94)	0.01	0.07
**Without depression**							
**flavodiet score (points)[1]**							
n= (cases)	17222 (116)	18295 (124)	16329 (118)	16990 (131)	17031 (125)		
HR (95% CI)	1.00	0.88 (0.68, 1.13)	0.89 (0.69, 1.16)	0.92 (0.71, 1.20)	0.85 (0.65, 1.11)	0.40	-
**Flavanones (mg/d)**							
n= (cases)	17174 (120)	17173 (111)	17174 (118)	17173 (121)	17173 (144)		
HR (95% CI)	1.00	0.89 (0.69, 1.15)	0.92 (0.71, 1.20)	0.90 (0.70, 1.17)	1.03 (0.80, 1.32)	0.73	-
**Anthocyanins (mg/d)**							
n= (cases)	17174 (116)	17173 (122)	17174 (131)	17173 (117)	17173 (128)		
HR (95% CI)	1.00	0.88 (0.68, 1.13)	0.87 (0.68, 1.13)	0.74 (0.56, 0.96)	0.75 (0.57, 0.97)	0.01	-

© 2024 Jennings A et al. *JAMA Network Open*.

**Flavan-3-ols (mg/d)**							
n= (cases)	17174 (123)	17173 (119)	17174 (135)	17173 (106)	17173 (131)		
HR (95% CI)	1.00	0.87 (0.67, 1.12)	0.95 (0.74, 1.22)	0.73 (0.56, 0.95)	0.88 (0.69, 1.14)	0.18	-
**Flavonols (mg/d)**							
n= (cases)	17174 (132)	17173 (119)	17174 (102)	17173 (134)	17173 (127)		
HR (95% CI)	1.00	0.80 (0.63, 1.03)	0.66 (0.51, 0.85)	0.83 (0.65, 1.07)	0.76 (0.59, 0.99)	0.10	-
**Flavones (mg/d)**							
n= (cases)	17174 (120)	17173 (117)	17174 (130)	17173 (138)	17173 (109)		
HR (95% CI)	1.00	0.85 (0.66, 1.10)	0.89 (0.69, 1.15)	0.92 (0.71, 1.20)	0.70 (0.53, 0.93)	0.06	-
**Polymers (mg/d)**							
n= (cases)	17174 (115)	17173 (114)	17174 (129)	17173 (132)	17173 (124)		
HR (95% CI)	1.00	0.85 (0.66, 1.11)	0.94 (0.73, 1.22)	0.93 (0.72, 1.20)	0.85 (0.65, 1.11)	0.45	-
**Proanthocyanidins (mg/d)** [2]							
n= (cases)	17174 (117)	17173 (116)	17174 (115)	17173 (130)	17173 (136)		
HR (95% CI)	1.00	0.87 (0.67, 1.12)	0.79 (0.61, 1.03)	0.84 (0.65, 1.10)	0.84 (0.64, 1.10)	0.25	-
**Total flavonoids (mg/d)**							
n= (cases)	17174 (124)	17173 (111)	17174 (127)	17173 (126)	17173 (126)		
HR (95% CI)	1.00	0.78 (0.60, 1.01)	0.85 (0.66, 1.10)	0.80 (0.62, 1.03)	0.80 (0.61, 1.03)	0.16	-

Values are adjusted hazard ratios (95% CI). [1] flavodiet score was calculated by summing intakes (in servings per day) of tea (black and green), red wine, apples, berries, grapes, oranges, grapefruit, sweet peppers, onions, and dark chocolate. [2]Proanthocyanidins are also included in the polymer subclass. Models adjusted for sex (male or female), socioeconomic status (Townsend Index, categorised as low [quintile 1], moderate [quintiles 2–4], high [quintile 5] deprivation), highest level of education (higher, vocational, upper Secondary, lower Secondary, none or unknown), ethnicity (White, Mixed, Asian, Black, Chinese, other group, not answered), current smoking status (yes or no), typical sleep duration (≤ 6 hours, 7–8 hours or > 8 hours, not answered), physical activity (total excess metabolic equivalents, in quintiles), BMI (kg/m^2), family history of dementia (yes or no), history of stroke (yes or no), post-menopausal status (yes, no, male or unknown), number of medications taken (1-3, 4-6, 7-9 or 10+), number of long-term health conditions (1-2, 3, 4, 5+), number of dietary assessments completed with plausible energy intakes (2, 3, 4 or 5), healthy plant-based diet index (score, in quintiles), alcohol intake not from red wine (g/d, in quintiles), energy intake (kcal/d, in quintiles) and fat intake (g/d, in quintiles) and stratified by region (London, North-West England, North-East England, Yorkshire, West Midlands, East Midlands, South-East England, South-West England, Scotland and Wales). P-interaction was calculated using all participants and refers to the interaction term between intake and indicator of hypertension (yes vs no) in the highest quintile of intake.

© 2024 Jennings A et al. *JAMA Network Open*.

eTable 9. Risk of Dementia by Quintiles of Flavodiet Score and Flavonoid Subclass Intake in 121 981 Males and Females From the UK Biobank, Stratified by Hypertension Status

Intake component	Q1	Q2	Q3	Q4	Q5	P-trend	P-interaction
**Hypertensive**							
**flavodiet score (points)[1]**							
n= (cases)	10855 (110)	10661 (107)	10795 (110)	10770 (112)	10613 (94)		
HR (95% CI)	1.00	0.86 (0.66, 1.13)	0.84 (0.64, 1.10)	0.84 (0.64, 1.10)	0.70 (0.52, 0.94)	0.03	0.51
**Flavanones (mg/d)**							
n= (cases)	10739 (112)	10739 (115)	10739 (92)	10739 (106)	10738 (108)		
HR (95% CI)	1.00	1.02 (0.78, 1.33)	0.77 (0.58, 1.02)	0.87 (0.66, 1.14)	0.85 (0.65, 1.11)	0.12	0.20
**Anthocyanins (mg/d)**							
n= (cases)	10739 (99)	10739 (102)	10739 (114)	10739 (111)	10738 (107)		
HR (95% CI)	1.00	0.86 (0.65, 1.14)	0.91 (0.69, 1.20)	0.84 (0.64, 1.12)	0.81 (0.61, 1.08)	0.18	0.75
**Flavan-3-ols (mg/d)**							
n= (cases)	10739 (117)	10739 (104)	10739 (120)	10739 (91)	10738 (101)		
HR (95% CI)	1.00	0.77 (0.59, 1.00)	0.86 (0.66, 1.11)	0.65 (0.49, 0.86)	0.71 (0.54, 0.93)	0.01	0.87
**Flavonols (mg/d)**							
n= (cases)	10739 (129)	10739 (104)	10739 (94)	10739 (102)	10738 (104)		
HR (95% CI)	1.00	0.73 (0.56, 0.94)	0.62 (0.48, 0.82)	0.66 (0.50, 0.86)	0.66 (0.50, 0.87)	<0.01	0.37
**Flavones (mg/d)**							
n= (cases)	10739 (111)	10739 (110)	10739 (112)	10739 (107)	10738 (93)		
HR (95% CI)	1.00	0.84 (0.65, 1.10)	0.85 (0.65, 1.11)	0.81 (0.61, 1.07)	0.69 (0.51, 0.93)	0.03	0.41
**Polymers (mg/d)**							
n= (cases)	10739 (106)	10739 (104)	10739 (122)	10739 (104)	10738 (97)		
HR (95% CI)	1.00	0.84 (0.63, 1.10)	0.97 (0.74, 1.26)	0.79 (0.60, 1.05)	0.73 (0.55, 0.97)	0.04	0.50
**Proanthocyanidins (mg/d)[2]**							
n= (cases)	10739 (106)	10739 (110)	10739 (103)	10739 (110)	10738 (104)		
HR (95% CI)	1.00	0.89 (0.68, 1.17)	0.77 (0.58, 1.02)	0.80 (0.60, 1.06)	0.72 (0.54, 0.98)	0.03	0.43
**Total flavonoids (mg/d)**							
n= (cases)	10739 (113)	10739 (102)	10739 (116)	10739 (102)	10738 (100)		
HR (95% CI)	1.00	0.77 (0.59, 1.02)	0.83 (0.64, 1.08)	0.72 (0.54, 0.94)	0.70 (0.53, 0.93)	0.01	0.98
**Non-hypertensive**							
**flavodiet score (points)[1]**							
n= (cases)	13928 (71)	13773 (62)	13693 (65)	13599 (81)	13294 (70)		
HR (95% CI)	1.00	0.75 (0.53, 1.05)	0.77 (0.54, 1.08)	0.92 (0.66, 1.28)	0.79 (0.55, 1.12)	0.54	-
**Flavanones (mg/d)**							
n= (cases)	13658 (64)	13657 (59)	13658 (70)	13657 (70)	13657 (86)		
HR (95% CI)	1.00	0.89 (0.62, 1.27)	1.05 (0.75, 1.49)	0.98 (0.69, 1.38)	1.16 (0.83, 1.62)	0.27	-
**Anthocyanins (mg/d)**							
n= (cases)	13658 (64)	13657 (70)	13658 (73)	13657 (71)	13657 (71)		
HR (95% CI)	1.00	0.89 (0.63, 1.25)	0.84 (0.59, 1.18)	0.76 (0.54, 1.09)	0.69 (0.48, 0.99)	0.03	-

© 2024 Jennings A et al. *JAMA Network Open*.

**Flavan-3-ols (mg/d)**							
n= (cases)	13658 (76)	13657 (72)	13658 (64)	13657 (74)	13657 (63)		
HR (95% CI)	1.00	0.89 (0.64, 1.24)	0.73 (0.52, 1.02)	0.83 (0.60, 1.15)	0.70 (0.50, 0.99)	0.05	-
**Flavonols (mg/d)**							
n= (cases)	13658 (74)	13657 (63)	13658 (57)	13657 (82)	13657 (73)		
HR (95% CI)	1.00	0.77 (0.55, 1.08)	0.62 (0.44, 0.89)	0.90 (0.65, 1.25)	0.77 (0.54, 1.08)	0.39	-
**Flavones (mg/d)**							
n= (cases)	13658 (69)	13657 (56)	13658 (76)	13657 (82)	13657 (66)		
HR (95% CI)	1.00	0.70 (0.49, 1.01)	0.92 (0.66, 1.29)	0.94 (0.67, 1.32)	0.73 (0.50, 1.06)	0.47	-
**Polymers (mg/d)**							
n= (cases)	13658 (64)	13657 (66)	13658 (74)	13657 (77)	13657 (68)		
HR (95% CI)	1.00	0.93 (0.66, 1.32)	0.99 (0.70, 1.39)	0.96 (0.68, 1.35)	0.86 (0.60, 1.23)	0.50	-
**Proanthocyanidins (mg/d)** [2]							
n= (cases)	13658 (70)	13658 (58)	13657 (66)	13657 (76)	13657 (79)		
HR (95% CI)	1.00	0.73 (0.51, 1.04)	0.80 (0.56, 1.13)	0.86 (0.61, 1.21)	0.83 (0.58, 1.18)	0.63	-
**Total flavonoids (mg/d)**							
n= (cases)	13658 (75)	13657 (60)	13658 (71)	13657 (80)	13657 (63)		
HR (95% CI)	1.00	0.74 (0.52, 1.04)	0.80 (0.57, 1.11)	0.85 (0.62, 1.18)	0.67 (0.47, 0.95)	0.11	-

Values are adjusted hazard ratios (95% CI). [1] flavodiet score was calculated by summing intakes (in servings per day) of tea (black and green), red wine, apples, berries, grapes, oranges, grapefruit, sweet peppers, onions, and dark chocolate. [2]Proanthocyanidins are also included in the polymer subclass. Models adjusted for sex (male or female), socioeconomic status (Townsend Index, categorised as low [quintile 1], moderate [quintiles 2–4], high [quintile 5] deprivation), highest level of education (higher, vocational, upper Secondary, lower Secondary, none or unknown), ethnicity (White, Mixed, Asian, Black, Chinese, other group, not answered), current smoking status (yes or no), typical sleep duration (≤ 6 hours, 7–8 hours or > 8 hours, not answered), physical activity (total excess metabolic equivalents, in quintiles), BMI (kg/m^2), family history of dementia (yes or no), history of stroke (yes or no), post-menopausal status (yes, no, male or unknown), number of medications taken (1-3, 4-6, 7-9 or 10+), number of long-term health conditions (1-2, 3, 4, 5+), number of dietary assessments completed with plausible energy intakes (2, 3, 4 or 5), healthy plant-based diet index (score, in quintiles), alcohol intake not from red wine (g/d, in quintiles), energy intake (kcal/d, in quintiles) and fat intake (g/d, in quintiles) and stratified by region (London, North-West England, North-East England, Yorkshire, West Midlands, East Midlands, South-East England, South-West England, Scotland and Wales). P-interaction was calculated using all participants and refers to the interaction term between intake and indicator of hypertension (yes vs no) in the highest quintile of intake.

© 2024 Jennings A et al. *JAMA Network Open*.

eTable 10. Risk of Dementia by Quintiles of Flavodiet Score and Flavonoid Subclass Intake in 61 719 Males and Females Aged More Than 60 Years From the UK Biobank

Intake component	Q1	Q2	Q3	Q4	Q5	P-trend
**flavodiet score (points)[1]**						
n= (cases)	12794 (197)	12156 (147)	12906 (181)	12276 (153)	11587 (134)	
HR (95% CI)	1.00	0.77 (0.62, 0.95)	0.88 (0.72, 1.09)	0.80 (0.64, 0.99)	0.72 (0.57, 0.91)	0.02
**Flavanones (mg/d)**						
n= (cases)	12344 (175)	12344 (155)	12344 (159)	12344 (146)	12343 (177)	
HR (95% CI)	1.00	0.90 (0.72, 1.12)	0.91 (0.74, 1.14)	0.84 (0.67, 1.05)	1.01 (0.81, 1.25)	0.87
**Anthocyanins (mg/d)**						
n= (cases)	12344 (189)	12344 (171)	12344 (148)	12344 (158)	12343 (146)	
HR (95% CI)	1.00	0.88 (0.72, 1.09)	0.77 (0.61, 0.95)	0.81 (0.65, 1.00)	0.72 (0.57, 0.90)	<0.01
**Flavan-3-ols (mg/d)**						
n= (cases)	12344 (202)	12344 (162)	12344 (161)	12344 (141)	12343 (146)	
HR (95% CI)	1.00	0.79 (0.64, 0.97)	0.77 (0.63, 0.95)	0.69 (0.55, 0.86)	0.69 (0.55, 0.86)	<0.01
**Flavonols (mg/d)**						
n= (cases)	12344 (206)	12344 (157)	12344 (142)	12344 (157)	12343 (150)	
HR (95% CI)	1.00	0.74 (0.60, 0.91)	0.66 (0.53, 0.82)	0.73 (0.59, 0.90)	0.68 (0.54, 0.85)	<0.01
**Flavones (mg/d)**						
n= (cases)	12344 (189)	12344 (155)	12344 (168)	12344 (159)	12343 (141)	
HR (95% CI)	1.00	0.79 (0.64, 0.98)	0.84 (0.68, 1.04)	0.81 (0.65, 1.01)	0.69 (0.55, 0.88)	0.01
**Polymers (mg/d)**						
n= (cases)	12344 (184)	12344 (155)	12344 (184)	12344 (141)	12343 (148)	
HR (95% CI)	1.00	0.80 (0.64, 0.99)	0.96 (0.78, 1.18)	0.73 (0.58, 0.91)	0.75 (0.60, 0.94)	0.01
**Proanthocyanidins (mg/d)[2]**						
n= (cases)	12344 (195)	12344 (146)	12344 (153)	12344 (159)	12343 (159)	
HR (95% CI)	1.00	0.71 (0.57, 0.88)	0.74 (0.60, 0.93)	0.75 (0.60, 0.94)	0.72 (0.57, 0.90)	0.02
**Total flavonoids (mg/d)**						
n= (cases)	12344 (199)	12344 (148)	12344 (166)	12344 (147)	12343 (152)	
HR (95% CI)	1.00	0.72 (0.58, 0.90)	0.80 (0.65, 0.98)	0.71 (0.57, 0.88)	0.71 (0.57, 0.89)	0.01

Values are adjusted hazard ratios (95% CI), n=61,719 (812 cases). [1]flavodiet score was calculated by summing intakes (in servings per day) of tea (black and green), red wine, apples, berries, grapes, oranges, grapefruit, sweet peppers, onions, and dark chocolate. [2]Proanthocyanidins are also included in the polymer subclass. Models adjusted for sex (male or female), socioeconomic status (Townsend Index, categorised as low [quintile 1], moderate [quintiles 2–4], high [quintile 5] deprivation), highest level of education (higher, vocational, upper Secondary, lower Secondary, none or unknown), ethnicity (White, Mixed, Asian, Black, Chinese, other group, not answered), current smoking status (yes or no), typical sleep duration (≤ 6 hours, 7–8 hours or > 8 hours, not answered), physical activity (total excess metabolic equivalents, in quintiles), BMI (kg/m^2), family history of dementia (yes or no), history of stroke (yes or no), post-menopausal status (yes, no, male or unknown), number of medications taken (1-3, 4-6, 7-9 or 10+), number of long-term health conditions (1-2, 3, 4, 5+), number of dietary assessments completed with plausible energy intakes (2, 3, 4 or 5), healthy plant-based diet index (score, in quintiles), alcohol intake not from red wine (g/d, in quintiles), energy intake (kcal/d, in quintiles) and fat intake (g/d, in quintiles) and stratified by region (London, North-West England, North-East England, Yorkshire, West Midlands, East Midlands, South-East England, South-West England, Scotland and Wales).

© 2024 Jennings A et al. *JAMA Network Open*.

eTable 11. Risk of Dementia by Quintiles of Flavodiet Score and Flavonoid Subclass Intake in 120 959 Males and Females With at Least 5 Years Follow-Up From the UK Biobank

Intake component	Q1	Q2	Q3	Q4	Q5	P-trend
**flavodiet score (points)[1]**						
n= (cases)	23044 (166)	23740 (154)	23787 (161)	24417 (189)	25971 (173)	
HR (95% CI)	1.00	0.79 (0.63, 0.98)	0.76 (0.61, 0.95)	0.87 (0.70, 1.08)	0.71 (0.56, 0.89)	0.03
**Flavanones (mg/d)**						
n= (cases)	24193 (165)	24192 (166)	24191 (159)	24192 (163)	24191 (190)	
HR (95% CI)	1.00	0.97 (0.78, 1.21)	0.90 (0.72, 1.12)	0.89 (0.71, 1.11)	0.99 (0.80, 1.23)	0.73
**Anthocyanins (mg/d)**						
n= (cases)	24193 (150)	24192 (167)	24191 (167)	24192 (180)	24191 (179)	
HR (95% CI)	1.00	0.92 (0.74, 1.15)	0.85 (0.68, 1.07)	0.86 (0.68, 1.07)	0.80 (0.64, 1.00)	0.05
**Flavan-3-ols (mg/d)**						
n= (cases)	24193 (186)	24192 (163)	24191 (181)	24192 (152)	24191 (161)	
HR (95% CI)	1.00	0.79 (0.64, 0.97)	0.82 (0.67, 1.01)	0.68 (0.55, 0.85)	0.71 (0.57, 0.89)	<0.01
**Flavonols (mg/d)**						
n= (cases)	24193 (185)	24192 (166)	24191 (143)	24192 (177)	24191 (172)	
HR (95% CI)	1.00	0.80 (0.64, 0.98)	0.64 (0.51, 0.80)	0.77 (0.62, 0.95)	0.72 (0.58, 0.90)	0.01
**Flavones (mg/d)**						
n= (cases)	24193 (171)	24192 (153)	24191 (181)	24192 (181)	24191 (157)	
HR (95% CI)	1.00	0.77 (0.62, 0.96)	0.88 (0.71, 1.09)	0.86 (0.69, 1.07)	0.73 (0.57, 0.92)	0.06
**Polymers (mg/d)**						
n= (cases)	24193 (161)	24192 (163)	24191 (185)	24192 (177)	24191 (157)	
HR (95% CI)	1.00	0.88 (0.71, 1.10)	0.96 (0.78, 1.19)	0.87 (0.70, 1.09)	0.76 (0.61, 0.96)	0.04
**Proanthocyanidins (mg/d)[2]**						
n= (cases)	24193 (169)	24192 (158)	24191 (166)	24192 (172)	24191 (178)	
HR (95% CI)	1.00	0.81 (0.65, 1.01)	0.79 (0.64, 0.99)	0.77 (0.62, 0.97)	0.76 (0.60, 0.96)	0.03
**Total flavonoids (mg/d)**						
n= (cases)	24193 (180)	24192 (153)	24191 (177)	24192 (172)	24191 (161)	
HR (95% CI)	1.00	0.75 (0.60, 0.93)	0.80 (0.65, 0.99)	0.75 (0.60, 0.92)	0.69 (0.56, 0.87)	<0.01

Values are adjusted hazard ratios (95% CI), n=120,959 (843 cases). [1] flavodiet score was calculated by summing intakes (in servings per day) of tea (black and green), red wine, apples, berries, grapes, oranges, grapefruit, sweet peppers, onions, and dark chocolate. [2]Proanthocyanidins are also included in the polymer subclass. Models adjusted for sex (male or female), socioeconomic status (Townsend Index, categorised as low [quintile 1], moderate [quintiles 2–4], high [quintile 5] deprivation), highest level of education (higher, vocational, upper Secondary, lower Secondary, none or unknown), ethnicity (White, Mixed, Asian, Black, Chinese, other group, not answered), current smoking status (yes or no), typical sleep duration (≤ 6 hours, 7–8 hours or > 8 hours, not answered), physical activity (total excess metabolic equivalents, in quintiles), BMI (kg/m^2), family history of dementia (yes or no), history of stroke (yes or no), post-menopausal status (yes, no, male or unknown), number of medications taken (1-3, 4-6, 7-9 or 10+), number of long-term health conditions (1-2, 3, 4, 5+), number of dietary assessments completed with plausible energy intakes (2, 3, 4 or 5), healthy plant-based diet index (score, in quintiles), alcohol intake not from red wine (g/d, in quintiles), energy intake (kcal/d, in quintiles) and fat intake (g/d, in quintiles) and stratified by region (London, North-West England, North-East England, Yorkshire, West Midlands, East Midlands, South-East England, South-West England, Scotland and Wales).

© 2024 Jennings A et al. *JAMA Network Open*.

eTable 12. Risk of Dementia by Quintiles of Flavodiet Score and Flavonoid Subclass Intake in 119 053 Males and Females With No Reported Stroke History From the UK Biobank

Intake component	Q1	Q2	Q3	Q4	Q5	P-trend
**flavodiet score (points)[1]**						
n= (cases)	24505 (163)	23148 (146)	24554 (165)	23082 (168)	23764 (146)	
HR (95% CI)	1.00	0.80 (0.64, 1.00)	0.81 (0.65, 1.02)	0.84 (0.68, 1.06)	0.71 (0.56, 0.90)	0.02
**Flavanones (mg/d)**						
n= (cases)	23811 (157)	23811 (152)	23811 (142)	23811 (158)	23809 (179)	
HR (95% CI)	1.00	0.93 (0.74, 1.17)	0.85 (0.67, 1.07)	0.90 (0.72, 1.13)	0.97 (0.78, 1.21)	0.75
**Anthocyanins (mg/d)**						
n= (cases)	23811 (139)	23811 (161)	23811 (159)	23811 (169)	23809 (160)	
HR (95% CI)	1.00	0.92 (0.73, 1.16)	0.87 (0.69, 1.09)	0.85 (0.67, 1.07)	0.75 (0.59, 0.95)	0.01
**Flavan-3-ols (mg/d)**						
n= (cases)	23811 (168)	23811 (156)	23811 (168)	23811 (150)	23809 (146)	
HR (95% CI)	1.00	0.83 (0.66, 1.03)	0.83 (0.67, 1.04)	0.73 (0.58, 0.91)	0.72 (0.57, 0.91)	<0.01
**Flavonols (mg/d)**						
n= (cases)	23811 (172)	23811 (156)	23811 (138)	23811 (162)	23809 (160)	
HR (95% CI)	1.00	0.80 (0.65, 1.00)	0.66 (0.53, 0.83)	0.74 (0.60, 0.93)	0.73 (0.58, 0.92)	0.01
**Flavones (mg/d)**						
n= (cases)	23811 (162)	23811 (150)	23811 (168)	23811 (167)	23809 (141)	
HR (95% CI)	1.00	0.80 (0.64, 1.00)	0.86 (0.69, 1.07)	0.82 (0.65, 1.03)	0.68 (0.53, 0.87)	0.01
**Polymers (mg/d)**						
n= (cases)	23811 (145)	23811 (158)	23811 (175)	23811 (166)	23809 (144)	
HR (95% CI)	1.00	0.94 (0.75, 1.18)	1.00 (0.80, 1.25)	0.90 (0.71, 1.13)	0.79 (0.62, 1.00)	0.05
**Proanthocyanidins (mg/d)[2]**						
n= (cases)	23811 (154)	23811 (147)	23811 (157)	23811 (161)	23809 (169)	
HR (95% CI)	1.00	0.82 (0.66, 1.04)	0.82 (0.65, 1.03)	0.80 (0.63, 1.01)	0.80 (0.63, 1.02)	0.10
**Total flavonoids (mg/d)**						
n= (cases)	23811 (164)	23811 (144)	23811 (167)	23811 (165)	23809 (148)	
HR (95% CI)	1.00	0.77 (0.61, 0.96)	0.83 (0.67, 1.03)	0.77 (0.62, 0.96)	0.71 (0.56, 0.89)	0.01

Values are adjusted hazard ratios (95% CI), n=119,053 (788 cases). [1] flavodiet score was calculated by summing intakes (in servings per day) of tea (black and green), red wine, apples, berries, grapes, oranges, grapefruit, sweet peppers, onions, and dark chocolate. [2]Proanthocyanidins are also included in the polymer subclass. Models adjusted for sex (male or female), socioeconomic status (Townsend Index, categorised as low [quintile 1], moderate [quintiles 2–4], high [quintile 5] deprivation), highest level of education (higher, vocational, upper Secondary, lower Secondary, none or unknown), ethnicity (White, Mixed, Asian, Black, Chinese, other group, not answered), current smoking status (yes or no), typical sleep duration (≤ 6 hours, 7–8 hours or > 8 hours, not answered), physical activity (total excess metabolic equivalents, in quintiles), BMI (kg/m^2), family history of dementia (yes or no), post-menopausal status (yes, no, male or unknown), number of medications taken (1-3, 4-6, 7-9 or 10+), number of long-term health conditions (1-2, 3, 4, 5+), number of dietary assessments completed with plausible energy intakes (2, 3, 4 or 5), healthy plant-based diet index (score, in quintiles), alcohol intake not from red wine (g/d, in quintiles), energy intake (kcal/d, in quintiles) and fat intake (g/d, in quintiles) and stratified by region (London, North-West England, North-East England, Yorkshire, West Midlands, East Midlands, South-East England, South-West England, Scotland and Wales).

© 2024 Jennings A et al. *JAMA Network Open*.

eTable 13. Risk of Dementia by Quintiles of Flavodiet Score and Flavonoid Subclass Intake in 35 110 Males and Females At High Genetic Risk of Dementia Whose Reported Ethnicity Was White From the UK Biobank

Intake component	Q1	Q2	Q3	Q4	Q5	P-trend
**flavodiet score (points)[1]**						
n= (cases)	7128 (109)	7277 (86)	6854 (86)	7226 (96)	6625 (75)	
HR (95% CI)	1.00	0.64 (0.48, 0.86)	0.66 (0.50, 0.88)	0.69 (0.51, 0.91)	0.59 (0.43, 0.82)	0.01
**Flavanones (mg/d)**						
n= (cases)	7023 (90)	7022 (81)	7022 (90)	7022 (86)	7021 (105)	
HR (95% CI)	1.00	0.89 (0.65, 1.20)	0.93 (0.69, 1.25)	0.86 (0.64, 1.17)	0.99 (0.74, 1.33)	0.96
**Anthocyanins (mg/d)**						
n= (cases)	7023 (94)	7022 (84)	7022 (101)	7022 (93)	7021 (80)	
HR (95% CI)	1.00	0.72 (0.53, 0.97)	0.82 (0.62, 1.10)	0.71 (0.52, 0.95)	0.60 (0.44, 0.82)	<0.01
**Flavan-3-ols (mg/d)**						
n= (cases)	7023 (111)	7022 (90)	7022 (94)	7022 (82)	7021 (75)	
HR (95% CI)	1.00	0.70 (0.53, 0.93)	0.72 (0.55, 0.95)	0.61 (0.46, 0.82)	0.58 (0.43, 0.78)	<0.01
**Flavonols (mg/d)**						
n= (cases)	7023 (114)	7022 (96)	7022 (69)	7022 (87)	7021 (86)	
HR (95% CI)	1.00	0.71 (0.54, 0.94)	0.50 (0.37, 0.68)	0.61 (0.45, 0.81)	0.60 (0.44, 0.80)	<0.01
**Flavones (mg/d)**						
n= (cases)	7023 (91)	7022 (86)	7022 (95)	7022 (96)	7021 (84)	
HR (95% CI)	1.00	0.83 (0.61, 1.11)	0.86 (0.64, 1.15)	0.90 (0.66, 1.21)	0.78 (0.56, 1.08)	0.29
**Polymers (mg/d)**						
n= (cases)	7023 (98)	7022 (91)	7022 (99)	7022 (86)	7021 (78)	
HR (95% CI)	1.00	0.75 (0.56, 1.00)	0.84 (0.63, 1.12)	0.67 (0.50, 0.90)	0.63 (0.46, 0.86)	<0.01
**Proanthocyanidins (mg/d)[2]**						
n= (cases)	7023 (103)	7022 (92)	7022 (92)	7022 (80)	7021 (85)	
HR (95% CI)	1.00	0.78 (0.58, 1.03)	0.73 (0.55, 0.97)	0.60 (0.44, 0.81)	0.61 (0.44, 0.83)	<0.01
**Total flavonoids (mg/d)**						
n= (cases)	7023 (113)	7022 (80)	7022 (95)	7022 (85)	7021 (79)	
HR (95% CI)	1.00	0.59 (0.44, 0.79)	0.69 (0.52, 0.91)	0.58 (0.43, 0.78)	0.57 (0.42, 0.77)	<0.01

Values are adjusted hazard ratios (95% CI), n=35,110 (452 cases). [1] flavodiet score was calculated by summing intakes (in servings per day) of tea (black and green), red wine, apples, berries, grapes, oranges, grapefruit, sweet peppers, onions, and dark chocolate. [2]Proanthocyanidins are also included in the polymer subclass. Models adjusted for sex (male or female), socioeconomic status (Townsend Index, categorised as low [quintile 1], moderate [quintiles 2–4], high [quintile 5] deprivation), highest level of education (higher, vocational, upper Secondary, lower Secondary, none or unknown), ethnicity (White, Mixed, Asian, Black, Chinese, other group, not answered), current smoking status (yes or no), typical sleep duration (≤ 6 hours, 7–8 hours or > 8 hours, not answered), physical activity (total excess metabolic equivalents, in quintiles), BMI (kg/m^2), family history of dementia (yes or no), history of stroke (yes or no), post-menopausal status (yes, no, male or unknown), number of medications taken (1-3, 4-6, 7-9 or 10+), number of long-term health conditions (1-2, 3, 4, 5+), genetic kinship (at least one relative, no kinship, unknown), number of dietary assessments completed with plausible energy intakes (2, 3, 4 or 5), healthy plant-based diet index (score, in quintiles), alcohol intake not from red wine (g/d, in quintiles), energy intake (kcal/d, in quintiles) and fat intake (g/d, in quintiles) and stratified by region (London, North-West England, North-East England, Yorkshire, West Midlands, East Midlands, South-East England, South-West England, Scotland and Wales).

© 2024 Jennings A et al. *JAMA Network Open*.

eTable 14. Risk of Dementia by Quintiles of Flavodiet Score and Flavonoid Subclass Intake in 121 986 Males and Females From the UK Biobank Adjusted for Individual Foods Associated With Dementia Risk)

Intake component	Q1	Q2	Q3	Q4	Q5	P-trend
**flavodiet score (points)[1]**						
n= (cases)	23264 (173)	23944 (161)	24004 (173)	24605 (194)	26169 (181)	
HR (95% CI)	1.00	0.78 (0.63, 0.97)	0.78 (0.63, 0.97)	0.85 (0.68, 1.06)	0.71 (0.57, 0.90)	0.03
**Flavanones (mg/d)**						
n= (cases)	24399 (177)	24396 (171)	24397 (165)	24397 (171)	24397 (198)	
HR (95% CI)	1.00	0.95 (0.76, 1.17)	0.89 (0.72, 1.10)	0.89 (0.72, 1.10)	0.99 (0.81, 1.22)	0.79
**Anthocyanins (mg/d)**						
n= (cases)	24398 (160)	24397 (173)	24397 (179)	24397 (186)	24397 (184)	
HR (95% CI)	1.00	0.91 (0.73, 1.13)	0.88 (0.71, 1.09)	0.87 (0.70, 1.08)	0.82 (0.66, 1.03)	0.09
**Flavan-3-ols (mg/d)**						
n= (cases)	24398 (193)	24397 (171)	24397 (188)	24397 (163)	24397 (167)	
HR (95% CI)	1.00	0.78 (0.63, 0.96)	0.79 (0.64, 0.98)	0.68 (0.54, 0.85)	0.68 (0.54, 0.86)	<0.01
**Flavonols (mg/d)**						
n= (cases)	24398 (193)	24397 (175)	24397 (149)	24397 (186)	24397 (179)	
HR (95% CI)	1.00	0.80 (0.65, 0.99)	0.64 (0.51, 0.79)	0.77 (0.62, 0.95)	0.72 (0.58, 0.91)	0.01
**Flavones (mg/d)**						
n= (cases)	24398 (181)	24397 (164)	24397 (189)	24397 (187)	24397 (161)	
HR (95% CI)	1.00	0.80 (0.65, 1.00)	0.90 (0.73, 1.11)	0.88 (0.72, 1.09)	0.76 (0.61, 0.95)	0.08
**Polymers (mg/d)**						
n= (cases)	24398 (169)	24397 (168)	24397 (195)	24397 (185)	24397 (165)	
HR (95% CI)	1.00	0.87 (0.70, 1.08)	0.96 (0.78, 1.19)	0.86 (0.69, 1.07)	0.75 (0.59, 0.95)	0.03
**Proanthocyanidins (mg/d)[2]**						
n= (cases)	24398 (176)	24397 (167)	24397 (171)	24397 (181)	24397 (187)	
HR (95% CI)	1.00	0.83 (0.67, 1.03)	0.79 (0.64, 0.99)	0.79 (0.64, 0.99)	0.79 (0.63, 0.99)	0.07
**Total flavonoids (mg/d)**						
n= (cases)	24398 (188)	24397 (156)	24397 (189)	24397 (179)	24397 (170)	
HR (95% CI)	1.00	0.73 (0.59, 0.90)	0.81 (0.66, 1.00)	0.73 (0.59, 0.91)	0.69 (0.54, 0.86)	0.01

Values are adjusted hazard ratios (95% CI), n=121,986 (882 cases). [1] flavodiet score was calculated by summing intakes (in servings per day) of tea (black and green), red wine, apples, berries, grapes, oranges, grapefruit, sweet peppers, onions, and dark chocolate. [2]Proanthocyanidins are also included in the polymer subclass. Models adjusted for sex (male or female), socioeconomic status (Townsend Index, categorised as low [quintile 1], moderate [quintiles 2–4], high [quintile 5] deprivation), highest level of education (higher, vocational, upper Secondary, lower Secondary, none or unknown), ethnicity (White, Mixed, Asian, Black, Chinese, other group, not answered), current smoking status (yes or no), typical sleep duration (≤ 6 hours, 7–8 hours or > 8 hours, not answered), physical activity (total excess metabolic equivalents, in quintiles), BMI (kg/m^2), family history of dementia (yes or no), history of stroke (yes or no), post-menopausal status (yes, no, male or unknown), number of medications taken (1-3, 4-6, 7-9 or 10+), number of long-term health conditions (1-2, 3, 4, 5+), number of dietary assessments completed with plausible energy intakes (2, 3, 4 or 5), intakes of red and processed meats, oily fish, coffee, high sugar foods and drinks, alcohol not from red wine, fat (all g/d, in quintiles), energy intake (kcal/d, in quintiles) and stratified by region (London, North-West England, North-East England, Yorkshire, West Midlands, East Midlands, South-East England, South-West England, Scotland and Wales).

© 2024 Jennings A et al. *JAMA Network Open*.

eTable 15. Risk of Dementia by Quintiles of Flavodiet Score and Flavonoid Subclass Intake in 53 101 Males and Females From the UK Biobank Living in High Areas of Deprivation or With Low Levels of Education

Intake component	Q1	Q2	Q3	Q4	Q5	P-trend
**flavodiet score (points)[1]**						
n= (cases)	10802 (88)	11322 (91)	9926 (81)	10613 (93)	10438 (85)	
HR (95% CI)	1.00	0.83 (0.62, 1.12)	0.78 (0.57, 1.06)	0.81 (0.60, 1.09)	0.74 (0.54, 1.02)	0.09
**Flavanones (mg/d)**						
n= (cases)	10621 (95)	10620 (89)	10620 (73)	10620 (79)	10620 (102)	
HR (95% CI)	1.00	0.87 (0.65, 1.17)	0.72 (0.53, 0.98)	0.72 (0.53, 0.98)	0.89 (0.67, 1.19)	0.26
**Anthocyanins (mg/d)**						
n= (cases)	10621 (77)	10620 (82)	10620 (96)	10620 (90)	10620 (93)	
HR (95% CI)	1.00	0.86 (0.63, 1.18)	0.93 (0.69, 1.27)	0.82 (0.60, 1.12)	0.82 (0.60, 1.13)	0.24
**Flavan-3-ols (mg/d)**						
n= (cases)	10621 (93)	10620 (86)	10620 (101)	10620 (76)	10620 (82)	
HR (95% CI)	1.00	0.83 (0.61, 1.11)	0.89 (0.67, 1.18)	0.66 (0.49, 0.90)	0.69 (0.51, 0.94)	0.01
**Flavonols (mg/d)**						
n= (cases)	10621 (95)	10620 (85)	10620 (80)	10620 (91)	10620 (87)	
HR (95% CI)	1.00	0.80 (0.59, 1.07)	0.68 (0.50, 0.92)	0.75 (0.56, 1.01)	0.69 (0.51, 0.94)	0.03
**Flavones (mg/d)**						
n= (cases)	10621 (84)	10620 (94)	10620 (93)	10620 (86)	10620 (81)	
HR (95% CI)	1.00	0.94 (0.70, 1.27)	0.86 (0.63, 1.17)	0.81 (0.59, 1.12)	0.77 (0.55, 1.08)	0.09
**Polymers (mg/d)**						
n= (cases)	10621 (83)	10620 (83)	10620 (98)	10620 (93)	10620 (81)	
HR (95% CI)	1.00	0.91 (0.67, 1.24)	0.98 (0.73, 1.32)	0.86 (0.64, 1.16)	0.74 (0.54, 1.02)	0.06
**Proanthocyanidins (mg/d)[2]**						
n= (cases)	10621 (88)	10620 (79)	10620 (83)	10620 (89)	10620 (99)	
HR (95% CI)	1.00	0.76 (0.56, 1.04)	0.75 (0.55, 1.02)	0.75 (0.55, 1.02)	0.81 (0.59, 1.11)	0.27
**Total flavonoids (mg/d)**						
n= (cases)	10621 (91)	10620 (80)	10620 (95)	10620 (91)	10620 (81)	
HR (95% CI)	1.00	0.79 (0.58, 1.07)	0.84 (0.63, 1.13)	0.76 (0.57, 1.03)	0.66 (0.49, 0.91)	0.02

Values are adjusted hazard ratios (95% CI) in participants living in areas of high deprivation [Townsend Index quintile 5] (n= 24,309) or with low levels of education [highest level of education lower Secondary or none] (n=28,792), n=53,101 (438 cases). [1] flavodiet score was calculated by summing intakes (in servings per day) of tea (black and green), red wine, apples, berries, grapes, oranges, grapefruit, sweet peppers, onions, and dark chocolate. [2]Proanthocyanidins are also included in the polymer subclass. Models adjusted for sex (male or female), ethnicity (White, Mixed, Asian, Black, Chinese, other group, not answered), current smoking status (yes or no), typical sleep duration (≤ 6 hours, 7–8 hours or > 8 hours, not answered), physical activity (total excess metabolic equivalents, in quintiles), BMI (kg/m^2), family history of dementia (yes or no), history of stroke (yes or no), post-menopausal status (yes, no, male or unknown), number of medications taken (1-3, 4-6, 7-9 or 10+), number of long-term health conditions (1-2, 3, 4, 5+), number of dietary assessments completed with plausible energy intakes (2, 3, 4 or 5), intakes of red and processed meats, oily fish, coffee, high sugar foods and drinks, alcohol not from red wine, fat (all g/d, in quintiles), energy intake (kcal/d, in quintiles) and stratified by region (London, North-West England, North-East England, Yorkshire, West Midlands, East Midlands, South-East England, South-West England, Scotland and Wales).

© 2024 Jennings A et al. *JAMA Network Open*.

eTable 16. Risk of Dementia by Quintiles of Flavodiet Score and Flavonoid Subclass Intake in 73 200 Males and Females From the UK Biobank With Low or Moderate Levels of Physical Activity

Intake component	Q1	Q2	Q3	Q4	Q5	P-trend
**flavodiet score (points)[1]**						
n= (cases)	15516 (115)	14410 (105)	14941 (110)	14486 (131)	13847 (87)	
HR (95% CI)	1.00	0.84 (0.64, 1.10)	0.83 (0.64, 1.08)	0.97 (0.74, 1.25)	0.67 (0.50, 0.90)	0.07
**Flavanones (mg/d)**						
n= (cases)	14641 (102)	14640 (115)	14640 (109)	14640 (98)	14639 (124)	
HR (95% CI)	1.00	1.07 (0.81, 1.40)	0.97 (0.74, 1.28)	0.83 (0.63, 1.10)	1.01 (0.77, 1.33)	0.50
**Anthocyanins (mg/d)**						
n= (cases)	14641 (100)	14640 (108)	14640 (108)	14640 (117)	14639 (115)	
HR (95% CI)	1.00	0.90 (0.68, 1.19)	0.82 (0.62, 1.08)	0.83 (0.63, 1.10)	0.76 (0.57, 1.01)	0.06
**Flavan-3-ols (mg/d)**						
n= (cases)	14641 (124)	14640 (114)	14640 (104)	14640 (102)	14639 (104)	
HR (95% CI)	1.00	0.82 (0.64, 1.06)	0.72 (0.55, 0.93)	0.70 (0.54, 0.92)	0.71 (0.54, 0.92)	0.01
**Flavonols (mg/d)**						
n= (cases)	14641 (125)	14640 (101)	14640 (90)	14640 (124)	14639 (108)	
HR (95% CI)	1.00	0.72 (0.55, 0.94)	0.60 (0.45, 0.79)	0.83 (0.64, 1.08)	0.70 (0.53, 0.92)	0.07
**Flavones (mg/d)**						
n= (cases)	14641 (108)	14640 (103)	14640 (116)	14640 (119)	14639 (102)	
HR (95% CI)	1.00	0.81 (0.62, 1.07)	0.88 (0.67, 1.15)	0.87 (0.67, 1.15)	0.75 (0.56, 1.01)	0.17
**Polymers (mg/d)**						
n= (cases)	14641 (115)	14640 (102)	14640 (117)	14640 (112)	14639 (102)	
HR (95% CI)	1.00	0.79 (0.60, 1.03)	0.88 (0.67, 1.14)	0.79 (0.61, 1.04)	0.73 (0.55, 0.96)	0.05
**Proanthocyanidins (mg/d) [2]**						
n= (cases)	14641 (121)	14640 (98)	14640 (104)	14640 (113)	14639 (112)	
HR (95% CI)	1.00	0.71 (0.54, 0.93)	0.71 (0.54, 0.92)	0.71 (0.54, 0.93)	0.68 (0.52, 0.91)	0.02
**Total flavonoids (mg/d)**						
n= (cases)	14641 (120)	14640 (104)	14640 (108)	14640 (114)	14639 (102)	
HR (95% CI)	1.00	0.78 (0.59, 1.01)	0.75 (0.58, 0.98)	0.76 (0.59, 0.99)	0.69 (0.53, 0.92)	0.02

Values are adjusted hazard ratios (95% CI) in participants with low or moderate levels of physical activity [total excess metabolic equivalents, quintiles 1-3], n=73,200 (548 cases). [1] flavodiet score was calculated by summing intakes (in servings per day) of tea (black and green), red wine, apples, berries, grapes, oranges, grapefruit, sweet peppers, onions, and dark chocolate. [2]Proanthocyanidins are also included in the polymer subclass. Models adjusted for sex (male or female), socioeconomic status (Townsend Index, categorised as low [quintile 1], moderate [quintiles 2–4], high [quintile 5] deprivation), highest level of education (higher, vocational, upper Secondary, lower Secondary, none or unknown), ethnicity (White, Mixed, Asian, Black, Chinese, other group, not answered), current smoking status (yes or no), typical sleep duration (≤ 6 hours, 7–8 hours or > 8 hours, not answered), BMI (kg/m^2), family history of dementia (yes or no), history of stroke (yes or no), post-menopausal status (yes, no, male or unknown), number of medications taken (1-3, 4-6, 7-9 or 10+), number of long-term health conditions (1-2, 3, 4, 5+), number of dietary assessments completed with plausible energy intakes (2, 3, 4 or 5), intakes of red and processed meats, oily fish, coffee, high sugar foods and drinks, alcohol not from red wine, fat (all g/d, in quintiles), energy intake (kcal/d, in quintiles) and stratified by region (London, North-West England, North-East England, Yorkshire, West Midlands, East Midlands, South-East England, South-West England, Scotland and Wales).

© 2024 Jennings A et al. *JAMA Network Open*.

eReferences

1. Thompson AS, Tresserra-Rimbau A, Karavasiloglou N, et al. Association of Healthful Plant-based Diet Adherence With Risk of Mortality and Major Chronic Diseases Among Adults in the UK. *JAMA Netw Open*. Mar 1 2023;6(3):e234714. doi:10.1001/jamanetworkopen.2023.4714
2. Bycroft C, Freeman C, Petkova D, et al. The UK Biobank resource with deep phenotyping and genomic data. *Nature*. 2018/10/01 2018;562(7726):203-209. doi:10.1038/s41586-018-0579-z
3. Thompson D, Wells D, Selzam S, et al. UK Biobank release and systematic evaluation of optimised polygenic risk scores for 53 diseases and quantitative traits. medRxiv; 2022.
4. Lourida I, Hannon E, Littlejohns TJ, et al. Association of Lifestyle and Genetic Risk With Incidence of Dementia. *JAMA : the journal of the American Medical Association*. Jul 14 2019;doi:10.1001/jama.2019.9879
5. de Lange A-MG, Kaufmann T, Quintana DS, et al. Prominent health problems, socioeconomic deprivation, and higher brain age in lonely and isolated individuals: A population-based study. *Behavioural Brain Research*. 2021/09/24/ 2021;414:113510. doi:https://doi.org/10.1016/j.bbr.2021.113510

© 2024 Jennings A et al. *JAMA Network Open*.
