# Supplementary material for: Flavonoid-Rich Foods, Dementia Risk, and Interactions With Genetic Risk, Hypertension, and Depression
Source: JAMA Netw Open. 2024 Sep 18;7(9):e2434136. doi: 10.1001/jamanetworkopen.2024.34136 (PMC11411383; doi:10.1001/jamanetworkopen.2024.34136)
Supplement: Supplement 2. — Data Sharing Statement [file jamanetwopen-e2434136-s002.pdf]

Data Sharing Statement

Jennings. Flavonoid-Rich Foods, Dementia Risk, and Interactions With Genetic Risk, Hypertension, and Depression. *JAMA Netw Open*. Published September 18, 2024. doi:10.1001/jamanetworkopen.2024.34136

Data
Data available: No

Additional Information
Explanation for why data not available: UK Biobank data can be requested by all bona fide researchers for approved projects, including replication, through https://www.ukbiobank.ac.uk/.
